# "Therapeutic advancements in nanomedicine: The multifaceted roles of silver nanoparticles"

**DOI:** 10.1016/j.biotno.2024.05.002

**Published:** 2024-06-01

**Authors:** Karthik K Karunakar, Binoy Varghese Cheriyan, Krithikeshvaran R, Gnanisha M, Abinavi B

**Affiliations:** a Department of Pharmacy Practice, Saveetha College of Pharmacy, Saveetha Institute of Medical and Technical Sciences, Chennai, 602105, TN, India; b Department of Pharmaceutical Chemistry, Saveetha College of Pharmacy, Saveetha Institute of Medical and Technical Sciences, Chennai, 602105, TN, India

**Keywords:** Nanotechnology, AgNps, Biomedical applications, Anti-inflammation, Reactive oxygen species, Anticancer

## Abstract

Nanotechnology has the advantages of enhanced bioactivity, reduced toxicity, target specificity, and sustained release and NPs can penetrate cell membranes. The small size of silver nanoparticles, AgNPs, large surface area, and unique physicochemical properties contribute to cell lysis and increased permeability of cell membranes used in the field of biomedicine. Functional precursors integrate with phytochemicals to create distinctive therapeutic properties and the stability of the nanoparticles can be enhanced by Surface coatings and encapsulation methods, The current study explores the various synthesis methods and characterization techniques of silver nanoparticles (AgNPs) and highlights their intrinsic activity in therapeutic applications, Anti-cancer activity noted at a concentration range of 5–50 μg/ml and angiogenesis is mitigated at a dosage range of 10–50 μg/ml, Diabetes is controlled within the same concentration. Wound healing is improved at concentrations of 10–50 μg/ml and with a typical range of 10–08 μg/ml for bacteria with antimicrobial capabilities. Advancement of silver nanoparticles with a focus on the future use of AgNPs-coated wound dressings and medical devices to decrease the risk of infection. Chemotherapeutic drugs can be administered by AgNPs, which reduces adverse effects and an improvement in treatment outcomes. AgNPs have been found to improve cell proliferation and differentiation, making them beneficial for tissue engineering and regenerative medicine. Our study highlights emerging patterns and developments in the field of medicine, inferring potential future paths.

## Key Highlights

• Silver Nanoparticles (AgNPs), Bio-inspired, Bioreduction.
• Medicine extensively utilizes nanotechnology for precise medication delivery systems. Nanoparticles have generated considerable attention for medication delivery.
• Silver nanoparticles, which are small in size and possess powerful antibacterial capabilities, have been intensively examined for their capacity to encapsulate and deliver a variety of therapeutic agents.
• Harnessing AgNPs for Anti-Cancer, Anti-Angiogenic, and Anti-Diabetic Therapies, against HIV-1, MRSA.
• Future Perspectives: Emerging Trends in Silver Nanoparticle Biomedical Applications.

## Graphical abstract

**Figure:**
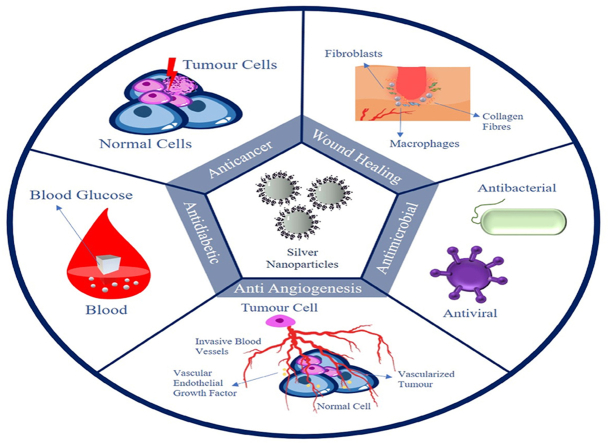

**Table:** Notations and Nomenclatures

Notation/Abbreviation	Full form
NPs	Nanoparticles
AgNPs	Silver Nanoparticles
UV–Vis	Ultraviolet–Visible
ROS	Reactive Oxygen Species
XRD	X-Ray Diffraction
FTIR	Fourier Transform Infrared Spectroscopy
SEM	Scanning Electron Microscopy
TEM	Transmission Electron Microscopy
DLS	Dynamic Light Scattering
EPR	Enhanced Permeability and Retention
MTT	3-(4,5-dimethylthiazol-2-yl)-2,5-diphenyltetrazolium bromide
PRP	Platelet-Rich Plasma
ECM	Extracellular Matrix
MMPs	Matrix Metalloproteinase
VEGF	Vascular Endothelial Growth Factor
NF-kB	Nuclear Factor kappa-light-chain-enhancer of activated B cells
COX-2	Cyclooxygenase-2
EGFR	Epidermal Growth Factor Receptor
DM	Diabetes Mellitus
AAI	Alpha-Amylase Inhibition
AGI	Alpha-Glucosidase Inhibition
STZ	Streptozotocin
MRSA	Methicillin-Resistant *Staphylococcus aureus*
VRE	Vancomycin-Resistant Enterococcus
HSV	Herpes Simplex Virus
HPIV	Human Parainfluenza Virus
HBV	Human Parainfluenza Virus
HIV-1	Human Immunodeficiency Virus Type 1
RSV	Respiratory Syncytial Virus
MIC	Minimum Inhibitory Concentration
PVP	Polyvinylpyrrolidone
AML	Acute Myeloid Leukemia
PI3K	Phosphoinositide 3-Kinase
PKA	cAMP-dependent Protein Kinase

## 1 Introduction

Nanotechnology has emerged as a significant field in the past 30 years, Nanotechnology has undergone a rapid rise in importance and currently has a wide-ranging impact on several fields, such as healthcare, cosmetics, biomedicine, food production, gene delivery, environmental preservation, mechanics, optics, and chemical industries.^1^ The collaborative endeavors in the field of nanomedicine have resulted in the development of new nanomaterials, particularly nanoparticles, and groundbreaking nanotherapeutics, Metallic nanoparticles (NPs) display a wide array of characteristics, and their size and surface activities contribute to a single dimension ranging from 1 to 100 nm.^2^ The combination of nanotechnology and medical practice has resulted in the emergence of an appealing topic called nanomedicine this discipline has become the subject of enormous research and has attracted significant interest. The versatility of nanoparticles stems from their small size and high surface area, which find usage in fields as diverse as cosmetics, medical diagnostics, and treatments.^3^ There are many methods for creating nanoparticles, such as chemical, physical, and biological synthesis.^4^ Short analysis time, toxin removal capability, low energy consumption, and the production of ecologically and sustainably harmless nanoparticles led to the selection of biological synthesis of NPs using plants over various physical, chemical, and biological methods associated with nanoparticle fabrication. Many branches of science are curious about silver nanoparticles, because of their useful biological properties and appealing physicochemical properties, silver nanoparticles are of special interest in the field of nanomedicine. The production of AgNPs with controlled size and form has been the subject of several investigations, and a multitude of specialized synthetic methodologies, including physical, chemical, and biological procedures, have been developed.^5^ There are two main types of physical procedures: mechanical and vapor-based. Producing very pure silver nanoparticles [AgNPs] with a uniform size distribution is possible via physical manufacturing.^6^ The majority of the approaches that are currently being developed for creating silver nanoparticles involve chemical reduction using a variety of organic and inorganic reducers, electrochemical techniques, radiolysis, or physicochemical reduction. These methods also address issues related to crystal formation, stability, aggregation, shape, size, and size distribution.^7^ The physical and chemical procedures are both very costly and very dangerous, It is interesting to note that AgNPs made naturally exhibit remarkable stability, high return, and solvency. Furthermore, there are just a handful of synthetic approaches to producing AgNPs; in contrast, natural processes seem to be transparent, rapid, safe, dependable, and ecologically benign. Under optimum conditions for translational research, they can generate size and morphological data with high degrees of certainty.^8^ The use of plant parts such as bark, peel, callus, leaves, and rhizomes is prevalent in biological production, as are microorganisms such as bacteria, fungi, and algae.^9^ Various plant-based silver nanoparticles (AgNPs) have been synthesized because of their antibacterial, wound-healing, and anti-inflammatory characteristics as well as their remarkable physiological potential at low concentrations, silver is sometimes called dynamic.^10^ Silver is a non-organic antibacterial chemical that is safe and can eradicate around 650 different kinds of disease-causing microbes.^11^ Silver nanoparticles are distinct metallic nanoparticles that possess biological activity and exhibit antibacterial properties against bacteria, fungi, and viruses.^12^, ^13^, ^14^ To assess biocompatibility or toxicity, it is essential to conduct a thorough characterization that considers several characteristics such as shape, size, surface area, solubility, and size distribution. The unique physical and chemical features of AgNPs may impact their biological traits. The synthetic nanoparticles underwent analysis using various scientific methodologies, such as scanning electron microscopy [SEM], transmission electron microscopy [TEM], dynamic light scattering [DLS], X-ray photoelectron spectroscopy [XPS], atomic force microscopy [AFM], and Fourier transform infrared spectroscopy [FTIR].^15^, ^16^, ^17^ Conditions such as Cancer, diabetes, wound healing, and microbial infections are given attention because of their profound influence on public health, widespread occurrence, and huge economic costs. Cancer is a prominent factor contributing to mortality, necessitating intricate therapies as a result of the diverse nature of tumors and their resistance to therapy. Diabetes has a global impact on a large number of people, resulting in serious problems if not effectively controlled. Chronic wounds, which are frequently observed in older individuals and those with chronic illnesses, are susceptible to infections that can hinder the healing process and lead to serious complications. The emergence of antibiotic-resistant bacteria presents a worldwide health emergency, threatening the efficacy of current medications and adding complexity to the management of infections. Conventional treatments such as Chemotherapy frequently cause harm to both healthy and malignant cells, resulting in significantly adverse consequences. Drug resistance occurs when cancer cells acquire the ability to withstand the effects of commonly used medicines, hence diminishing their effectiveness. Accurate and ongoing monitoring of glucose levels can be challenging and may require invasive treatments. Administration of Insulin Regular administration of insulin injections exacerbates pain and hampers the management of blood glucose levels. Regarding Infection Control, individuals with chronic wounds, particularly those with diabetes, are highly susceptible to infections that cannot be effectively treated with conventional methods. Impaired tissue regeneration and insufficient healing capacity can impede the healing process, particularly in vulnerable populations such as the elderly and individuals with diabetes. Antibiotic resistance makes numerous conventional antibiotics ineffective against bacteria, posing a significant worldwide health problem. Developing medications with the ability to combat a wide range of bacteria, fungi, and viruses is a challenging task. Recently, there has been a surge of studies focused on the potential of AgNPs to inhibit the growth of cancer, AgNPs have shown potential in efficiently combating several forms of cancer, including glioblastoma, colorectal adenocarcinoma, prostate, hepatic, nasopharyngeal, cervical, breast, and lung cancers.^18^ AgNPs may be delivered with precision using various agents, which augments their efficacy. AgNPs produced from plants, namely *Azadirachta indica* and *Ceropegia junce*a, have anti-angiogenic properties in living organisms, indicating their potential use in treating illnesses characterized by excessive angiogenesis.^19^ Diabetes mellitus [DM] is a metabolic disorder marked by chronically elevated levels of blood sugar, Diabetes is primarily caused by cellular insulin resistance or insufficient insulin production to lower blood sugar levels, hypoglycemic medicines are often used to either stimulate insulin production or enhance cell sensitivity.^20^ AgNPs were shown to have a significant hypoglycemic impact in comparison to glibenclamide, a conventional antidiabetic medication.^21^ AgNPs have also drawn interest in wound repair,^17^ The use of silver nanoparticles for wound healing purposes has shown a substantial rise in recent years. Silver-based therapies have been developed and have shown enhanced efficacy compared to conventional dressings.^22^ Studies showed AgNPs have antibacterial activity against a variety of diseases,^23^ including resistant bacteria, making them indispensable in medical and healthcare applications.^14^ Silver nanoparticles can impede bacterial growth. Prior studies have shown the efficacy of AgNPs antagonists in treating a range of drug-resistant illnesses, the multilayer antibacterial action of silver enhances its effectiveness against multi-drug-resistant pathogens and greatly reduces the likelihood of infections developing resistance.^19^^,^^24^^,^^25^ In the realm of biomedicine, this review discusses the synthesis, characterization, and biological applications of silver nanoparticles (AgNPs).

## 2 Methods of synthesis of silver nanoparticles

### 2.1 Physical method

The physical method, produces pure nanoparticles without chemical additions, achieving high purity and consistent particle size distribution.^26^ The Physical, chemical, and biological synthesis, produces AgNPs by reducing the precursor salt.^27^^,^^28^ The study determined that the thermal decomposition of Ag ^+^ -oleate complexes results in the formation of monodispersed silver nanocrystallites, Jung et al. observed the steady formation of polydisperse nanoparticles on the surface of a basic compact ceramic heater that was kept at a constant temperature. This was attributed to the condensation and evaporation processes of the spherical silver nanoparticles, which did not show any tendency to aggregate.^29^^,^^30^ Laser ablation of the polyol process has recently been shown to make circular nanoparticles of different sizes.^31^ Tsuji et al. used laser ablation in water to fabricate nanoscale silver particles and compare the dimensions and production efficiency of colloidal particles generated by nanosecond and femtosecond pulses, respectively the production efficiency of femtosecond pulses dropped, resulting in fewer dispersed colloids. Additionally, the colloids displayed a narrow size distribution and showed resistance to aggregation.^32^ AgNPs may be synthesized using laser ablation and evaporation-condensation methods, resulting in high-purity yields without the need for dangerous chemicals. The process employs a gas-phase mechanism at atmospheric pressure to produce nanospheres inside the tube furnace, there is a tank containing a metal resource that is first vaporized into a carrier gas this allows for the ultimate creation of the nanospheres.^33^ The size, shape, and number of NPs may be modified by altering the configuration of the reaction equipment. However, the process of creating AgNPs by steam condensation in a tube furnace has various difficulties, Space consumes a significant amount of energy, elevates the temperature in the vicinity of the metal source, and requires a considerable duration to achieve thermal stability, In order to overcome these challenges, Jung et al. showed that a ceramic heater may effectively be used for the production of AgNPs with a high concentration.^30^ Nevertheless, the process of agglomeration might happen when capping agents are not used, leading to increased operating costs owing to significant technology and a longer duration of synthesis.^34^ Physical methods provide advantages such as rapidity, radiation as a reducing agent, and the absence of toxic chemicals. Despite this, they also have drawbacks like high energy consumption, low yield, contamination of solvents, and uneven distribution.^35^, ^36^, ^37^

### 2.2 Biological method

Presently, more research is underway to develop "green" or ecologically sustainable methods for synthesizing metallic nanoparticles. The use of microorganisms or plants in biological approaches has gained popularity in recent years owing to its cost-effectiveness and eco-friendliness. The plant-mediated synthesis approach is considered the optimal biological process.^38^ A variety of biological systems, including bacteria, fungi, and plant extracts are used in the biological method.^39^^,^^40^ Multiple studies have recorded the production of AgNPs by environmentally friendly, cost-effective, and biocompatible methods, without the need for toxic chemicals.^41^ This green chemistry approach included several microbes.^42^, ^43^, ^44^, ^45^, ^46^ The Verticillium fungal system was used to examine the methodology of AgNP production by green synthesis.^47^^,^^48^ The main idea is that Ag ^+^ ions are bound by electrostatic interactions with the negatively charged carboxylate groups of the enzymes this leads to the formation of Ag nuclei, which continue to increase as more Ag ^+^ ions are reduced.^48^^,^^49^ Bacteria often use nitrate as a crucial source of nitrogen^34^^,^^50^ the use of nitrate reductase as a reducing agent is crucial in the biological reduction of Ag ^+^ ions.^41^ These technologies use live organisms, cellular extracts, or biological agents that are usually referred to as biological "nano factories".^51^ Biological synthesis procedures provide an environmentally friendly, very adaptable, and hygienic approach for synthesizing nanoparticles using a diverse array of physiological components.^52^ Table 1 describes the various sources for green synthesis for silver nanoparticles.

**Table 1:** AgNPs synthesized from various plant extracts and algae cultures.

Species	Type	Plant Source	Hydrodynamic Size [nm]	References
Plants	Terminalia mantaly	Stem bark, Root, and leaves	11–83	53
	Pyrus communis L. cultivars	skins and Fruit pulp	110–190	54
	Cotyledon orbiculate	leaves	100–140	55
	Acacia catechu	powder	5–80	56
	Solanum lycopersicum L.	Tomato	2–50	
	Allium cepa	Onion	5–80	
	*Monstera deliciosa*	Leaves	100–300	57
Algae	Spirulina sp.	Algae cultures	13.85	
	Coelastrum sp.		19.2	58
	Botryococcus braunii		15.67	
	*Haematococcus pluvialis*	Whole cell	200–500	59

### 2.3 Chemical method

Chemical synthesis processes need less complex and more convenient equipment. Prior studies indicate that silver ions when exposed to a reducing agent, acquire electrons and transform into a metallic state this eventually leads to the formation of silver nanoparticles due to their affordability, AgNO3 is commonly employed as a silver salt in the chemical production of silver nanoparticles.^1^^,^^58^ The study utilized AgNO3 as the main precursor for generating silver nanoparticles Trisodium citrate and sodium borohydride were employed as stabilizers. Sodium borohydride was found to be a highly effective reducing agent for producing silver nanoparticles with sizes ranging from 5 to 20 nm on the other hand, trisodium citrate was identified as the most efficient reducing agent for creating silver nanoparticles in the range of 60–100 nm.^60^ Abbasi et al. have thoroughly examined the characteristics, production methods, and medical applications of AgNPs.^61^ Patil et al. demonstrated the synthesis of silver nanoparticles by using polyvinyl alcohol as a stabilizer and hydrazine hydrate as a reducing agent their findings revealed the successful production of spherical-shaped nanoparticles, which have significant potential applications in biotechnology and pharmaceutical research.^62^ The synthesis of metallic silver nanoparticles involves the use of sodium citrate, ascorbate, sodium, elemental hydrogen, and block copolymers.^63^ The use of polyvinylpyrrolidone [PVP] as a size regulator resulted in the production of silver nanoparticles with a size lower than 10 nm.^64^ Chemical synthesis generates a wide range of noxious and perilous byproducts, necessitating the employment of compounds that minimize toxicity in these processes.^61^ Chemical processes use many methods such as Sono-decomposition, thermal decomposition, lithography, electrochemical reduction, laser irradiation, cryochemical synthesis, laser ablation, and chemical reduction.^65^, ^66^, ^67^ Despite the benefits of chemical-reducing agents, such as their ease of production, affordability, and high production capacity, they pose a significant threat to living beings due to their highly destructive nature.^40^

## 3 Various characterization techniques

### 3.1 UV–vis spectrophotometry

UV–Vis spectrophotometry is often used for the analysis of metal nanoparticles, enabling the monitoring of their durability and manufacturing process over an extended period.^66^^,^^67^ The creation of a metallic nanoparticle leads to a prominent area with significant absorptions in the visible spectrum the absorption within the wavelength range of 200–800 nm is suitable for analyzing nanoparticles within a measurement range of 2–100 nm.^68^ The analysis and examination of a surface plasmon peak have been identified for various types of nanoparticles made of metals with sizes ranging from 2 to 100 nm using UV–Vis spectrophotometry, The stability of silver nanoparticles produced through biological methods has been investigated and verified to exhibit surface plasmon resonance at a similar wavelength.^8^

### 3.2 X-ray diffraction [XRD]

Studying molecular and crystal structures allows for the qualitative identification of different compounds,^69^ The precise measurement of chemical substances,^70^ the measurement of the degree of crystallinity,^71^ isomorphous substitutions,^72^ particle sizes,^73^ The commonly used analytical method called X-ray diffraction [XRD] has effectively handled several applications^74^ the resulting scattering pattern provides evidence of the existence of nanoparticles with a crystalline structure. The Debye-Scherrer equation is used to calculate the particle size by analyzing the width of the Bragg reflection in the XRD data.^75^ Therefore, XRD enables the assessment of the internal arrangement of silver nanoparticles.^73^, ^74^, ^75^, ^76^ Studies have shown that X-ray diffraction (XRD) is a very effective technique for analyzing nanoparticles.

### 3.3 Fourier transform infrared spectroscopy [FTIR]

FTIR, or Fourier Transform Infrared Spectroscopy, is a technique that utilizes infrared light to analyze the chemical composition of substances, this approach offers high levels of precision and accuracy in determining the molecular structure of components. FTIR spectroscopy can detect absorbance changes as small as 10^−3^, making it advantageous for variance spectroscopy. This enables the separation of the protein's basal consumption from its functionally active segments.^77^ During FTIR analysis, the sample is exposed to infrared light, where a portion of the energy is absorbed by the sample and the remaining energy passes through it. The spectra exhibit the typical absorption and transmission characteristics of the elements present in the sample. Fourier Transform Infrared (FTIR) is a cost-effective, simple, and non-destructive technique.^78^

### 3.4 Scanning electron microscopy [SEM]

Recently, advancements in nanotechnology and nanoscience have resulted in the development of many high-resolution microscopy techniques. These techniques use a very potent electron beam to investigate objects at extremely tiny scales, providing new insights into the field of nanotechnology.^76^^,^^77^ Scanning electron microscopy (SEM) is a surface scanning method used in electron microscopy it is capable of detecting different particle measurements, size ranges, nanoparticles, and the surface structure.^79^ Scanning Electron Microscopy (SEM) is an effective technique for examining the surface characteristics and structure of nanoparticles, as well as determining their dimensions at the microscale (10^−6^) and nanoscale (10^−9^).^80^ The surface of the nanoparticles is subjected to a high-energy electron beam generated by SEM, and the outcomes reveal the distinctive characteristics of the sample.^81^ An electron microscope is used to analyze the cellular structure and identify any changes that occur before and after treatment with nanoparticles.^82^

### 3.5 Transmission electron microscopy [TEM]

Transmission Electron Microscopy (TEM) is a very effective imaging technique that can accurately see the internal structure of materials at the nanoscale. This makes it an essential tool for identifying nanoparticles it may be used to ascertain the dimensions, morphology, and distribution of particles and/or grains.^83^ Transmission electron microscopy (TEM) has a resolution that is one thousand times higher than scanning electron microscopy (SEM), resulting in more precise and detailed pictures that provide more accurate data.^80^^,^^82^

### 3.6 Dynamic light scattering [DLS]

Dynamic light scattering (DLS) is a widely used technique for estimating the size of a molecule. It was used to classify the dimensions of nanoparticles.^84^ The DLS method is often used to detect and characterize nanoparticles.^85^^,^^86^ DLS, a non-destructive technology, may be used to assess the average size of nanoparticles dispersed in liquids.^87^

## 4 Biomedical applications

**Fig. 1:**
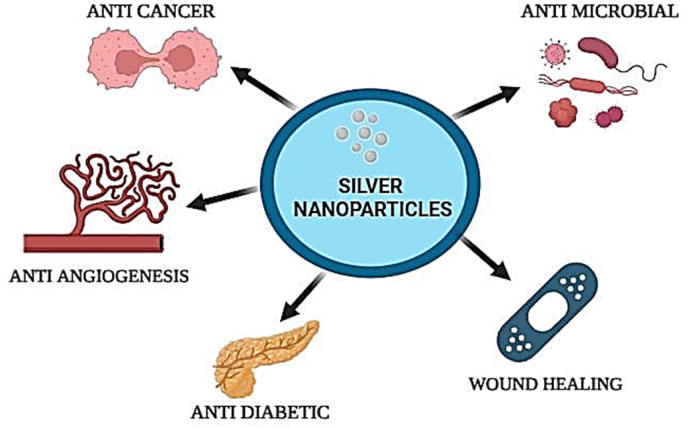
Multiple biological applications of silver nanoparticles (AgNPs).

AgNPs are intensively researched for diverse biological applications due to their outstanding features in contrast to elemental Ag, as seen in Fig. 1. The potent and wide-ranging antibacterial properties of silver nanoparticles (AgNPs) make them more desirable than other types of nanoparticles due to their ability to combat bacteria, fungi, and viruses because of their chemical stability and longevity, they continue to be effective for a long period.^88^ Silver nanoparticles (AgNPs) can be readily altered for specific applications and incorporated into materials to enhance their properties at low doses, they exhibit cytotoxicity reduction and cost reduction, as well as possess diverse mechanisms that decrease resistance.^89^ Silver nanoparticles (AgNPs) enhance the bioavailability of a chemical while minimizing its toxicity. The significant pro-oxidant and antioxidant effects provide new opportunities for research in a wide range of pathological conditions. AgNPs, or silver nanoparticles, possess anti-inflammatory, and immunomodulatory characteristics. These characteristics make them promising therapies for disorders associated with oxidative stress, inflammation, and downregulation of the immune system, all these conditions have been associated with diseases like cancer, angiogenesis, diabetes, antibiotic resistance, and wound healing. The present investigation offers a comprehensive analysis of the biological attributes of AgNPs and current progress in targeted strategies and novel applications.

### 4.1 Silver nanoparticles in cancer

Cancer is a very lethal disease characterized by the proliferation of aberrant cells that undergo uncontrolled division. The etiology of this condition is caused by genetic dysregulation or mutations resulting from acute or chronic exposure to medications or pollutants, In 2020 cancer remained the primary cause of death worldwide, with 18.1 million verified cases.^90^ Conventional cancer therapies include surgical intervention, radiation therapy, and chemotherapy, these drugs are often prescribed based on the pathological condition of the illness although there have been improvements in treatment methods, the survival rates of patients are still quite poor, and there is a high prevalence of adverse effects associated with chemotherapy, a significant number of chemotherapeutic medicines have restricted water solubility, Hydrophobic medicines have worse biocompatibility and hence need greater doses to reach therapeutic concentrations In addition, the limited solubility in water hinders the absorption of medication and leads to higher levels of toxicity in the body. Moreover, chemotherapy treatments have limited choice and inflict significant damage to healthy cells, leading to Adverse effects.^24^ Nanotechnology has made significant progress and is presently used in several fields, including medicine. Silver nanoparticles (AgNPs) possess inherent anticancer qualities using many mechanisms, including the release of silver ions or the production of free radicals these effects occur when AgNPs are assimilated by cell membranes, causing disruption to vital cellular processes and resulting in cell death and damage.^90^ Silver nanoparticles possess distinctive characteristics that make them a promising approach for cancer treatment from two perspectives Moreover, they may serve as vehicles for delivering anticancer medications, enabling the use of combination therapy, and they also exhibit inherent anticancer properties.^91^ Cancerous cells undergo apoptosis as a result of mitochondrial alterations, leading to their death,^92^ Disruptions in the levels of proteins that prevent cell death (antiapoptotic proteins) and enzymes that promote cell death (proapoptotic kinases),^93^ or the negative effects on structure and function caused by exposure to silver nanoparticles.^94^ Silver nanoparticles (AgNPs) may be continuously delivered to tumor tissues, where they accumulate in significant quantities the constant persistence is determined by the specific morphology of the cancer tissue,^95^ This results in the formation of an abnormal layer of cells lining the blood vessels, along with blood vessels that have narrow pores. The combination of these variables, along with complications with lymphocyte drainage, leads to the infiltration and buildup of small particles within malignant tissue. The phenomenon known as the enhanced permeability and retention (EPR) effect is currently utilized in nanotechnology for the development of medicines.^96^ Leukemia is a kind of cancer that originates in the bone marrow and leads to an overproduction of abnormal white blood cells, the research demonstrated the cytotoxic potential of PVP-coated silver nanoparticles against leukemic cells, specifically focusing on acute myeloid leukemia (AML) cells.^97^ The research found that the toxicity of silver nanoparticles is caused by the production of reactive oxygen species inside leukemic cells, the nanoparticles induced oxidative stress in the cells, leading to mitochondrial dysfunction and DNA damage.^98^ Seagrass extract was used as a bioreduction to synthesize silver nanoparticles with putative anticancer properties against A549 human lung cancer cells the efficacy of nanoparticles synthesized using seagrass extract as a bioreduction was assessed on cancer cells at concentrations ranging from 10 to 250 μg/ml the MTT (Microculture Tetrazolium Assay) assay demonstrated significant cytotoxic effects, leading to more than 80 % cell death at concentrations over 50 μg/ml The observed cytotoxic mechanism aligns with the findings of elevated caspase-3 synthesis, a gene that promotes programmed cell death.^99^

Vasantha et al. conducted a comprehensive investigation to ascertain the potential anticancer effects of environmentally friendly nano silver with the assistance of *Moringa oleifera* the researchers used many analytical approaches, such as high-vis spectrometry, resolution scanning electron microscopy, Fourier transform infrared spectroscopy, and atomic force microscopy [AFM], to identify the specific attributes of the silver nanoparticles.^100^ When exposed to a concentration of 250 μg/ml, silver nanoparticles induced a 94 % mortality rate in HeLa cells during 24 h, leading to the formation of membrane ulcerations, Morphological investigation at concentrations of 25, 50, and 100 μg/ml demonstrated the presence of apoptotic markers and cell segregation, resulting in necrosis.^101^ Moreover, the research revealed that inhibiting cell replication disrupted the cell cycle. In addition, the formation of reactive oxygen species (ROS) via the mitochondrial pathway was indirectly assessed, revealing a greater generation of hydrogen peroxide.^102^ Human lung cancer [A549] cells were subjected to testing using silver nanoparticles obtained from the extraction of *Juniperus chinensis* the study demonstrated that the presence of silver nanoparticles at a concentration of 9.87 μg/mL resulted in an increased activation of p53.^103^ When compared to cisplatin [CisPt] at a concentration of 24.67 μg/mL. Nanoparticles may induce chromatin condensation, nuclear fragmentation, and disintegration,^104^ perhaps by increased reactive oxygen species (ROS) generation,^105^ the level of p53 expression was seen to have increased Consequently, the presence of active p53 had an impact on the transcription and activity of genes that are involved in the cell cycle,^106^ As a result, there was a significant decrease in the levels of cyclin D1 and the number of cells in the G_0_/G_1_ phase, finally resulting in apoptosis.^107^ The presence of caspase-9 and executioner caspase-3 confirmed the occurrence of the apoptotic pathway in addition, the nanoparticles significantly decreased the concentrations of matrix metalloproteinases MMP-2 and MMP-9, which are associated with the processes of angiogenesis, invasiveness, and metastasis.^99^^,^^100^ Silver nanoparticles have been shown to have anticancer properties by several mechanisms they effectively engage with cancer cells by enhanced penetration and retention effect [EPR], enabling them to enter and accumulate inside the cells, resulting in cell death or inhibition of uncontrolled growth. Moreover, silver nanoparticles can modify signaling pathways, leading to the initiation of early cell death or the reduction of cancer cell growth Research indicates that silver nanoparticles stimulate proteins such as p53, caspase-3, and p-Er K_1/2_, resulting in apoptosis and the regulation of cell cycle.^101^^,^^102^

**Fig. 2:**
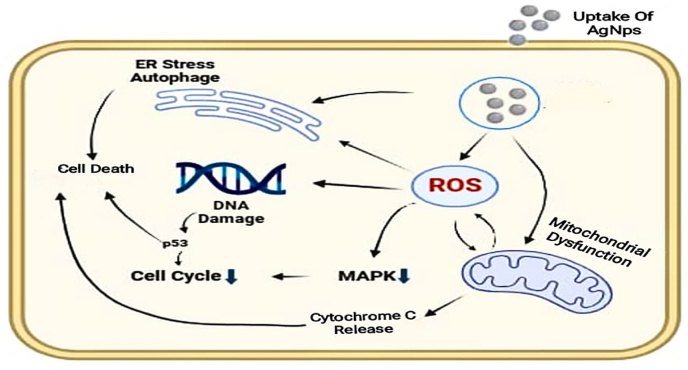
Illustrates the mechanism by which silver nanoparticles exhibit anti-cancer properties.

Miriam Buttacavoli et al. determined that the presence of silver nanoparticles had a positive impact on the growth and development of breast cancer cells, namely SKBR3 cells the researchers used contemporary methodologies and techniques to investigate the underlying mechanism of the anticancer effects of silver nanoparticles in human cancer cells their research provides a thorough comprehension of the inhibitory effects of silver nanoparticles on cell motility and metalloproteinases (MMPs).^108^ Observations reveal significant alterations in the morphology of cancer cells after treatment with silver nanoparticles, including cell shrinkage, irregular shape, cytoplasmic blebbing, modified intracellular vacuole shape, and chromatin condensation.^109^ The presence of reactive oxygen species led to oxidative damage and the death of cells In addition, they noted an elevation in the levels of LC3-II, ATG7, beclin-1, and ATG5, while seeing a decrease in HSP90.^110^ Our study focuses on the use of silver nanoparticles [AgNPs] in cancer therapy and their considerable pharmacological properties, supported by several studies. Silver nanoparticles (AgNPs) have demonstrated significant anticancer activity in vitro and in vivo, making them promising candidates for future research and therapeutic usage. Fig. 2 shows the significant mechanism of silver nanoparticles against cancer. AgNPs' capacity to induce apoptosis, impede cell proliferation, and disrupt the cell cycle makes them poisonous to cancer cells including leukemia, lung, and cervical cancer. These investigations highlight AgNPs' potential as new cancer therapies, notably their role in ROS-mediated anticancer effects. Table 2 lists the different cell line silver nanoparticle production techniques and their features. The average silver nanoparticle size is 50 nm. They penetrate capillaries in tissues and cells due to their tiny size and huge surface area. Chemically modifying their surfaces allows them to deliver a lot of medication. Surface oxidation, biomolecule conjugation or adhesion, and metallic ion discharge may affect AgNPs' cytotoxicity. Silver nanoparticles (AgNPs) might be utilized to develop novel, targeted cancer therapies that enhance patient outcomes and well-being.

**Table 2:** Various synthesis methods of silver nanoparticles in cell lines and their anti-cancer properties.

Green synthesis *Taraxacum officinale*	Liver hepatocellular carcinoma	HepG2	10–200 μg/mL dose-dependent	HepG2 observed a strong cytotoxic effect	^111^
Chemical biosynthesis Phycocyanin	Human breast adenocarcinoma	MCF-7	IC_50_:27.79 ± 2.3 μg/mL	inhibition of tumor growth in mice having Ehrlich-as cites carcinoma.	^112^
Mycosynthesis Inonotus obliquus	Human lung cancer human breast cancer	A549 and MCF7	IC_50_:100 μl/ml	A notable cytotoxic impact on the MCF-7 and A549 cell lines	^113^
Green Synthesis	Human breast cancer	MDA- MB-231	IC_50_:100 μg/ml	A significant apoptotic and cytotoxic effects	^114^
Acalypha indica Linn					
Green synthesis Sucrose	Malignant skin melanoma and squamous cell lung carcinoma	HT144 and H157	ID_50_: 3.6 μM	Remarkable antitumor potency compared to the clinically used reference compounds vincristine and methotrexate	^115^
Green synthesis	Breast Cancer	MDAMB- 231	IC_50_: 8,7 μg/mL	dose-dependent suppression of cellular proliferation	^116^
Ganoderma neo-japonicum Imazeki					
Chemical synthesis Polyvinylpyrrolidone [PVP]	Acute myeloid leukemia	AML	0–10 mg/mL	In six AML cells, AgNPs have been shown to exhibit an inhibitory impact with a low IC_50_ [0.90–3.43 mg/mL]. In HepG2, a significant cytotoxic effect was noted.	^98^
Green synthesis Commelina nudifloraL.	Colon Cancer	HCT-116	IC_50_:100 μg/mL	In HCT-116, decreased cell viability and elevated cytotoxic impact	^117^
Green synthesis Melia dubia	Human breast cancer	MCF-7	IC_50_:31.2 μl/ml	AgNps demonstrated exceptional cytotoxicity efficacy against MCF-7, as well as evidence of a high therapeutic index value.	^118^
Green synthesis Erythrina indica lam	Breast and lung cancer	MCF-7 and HEP G2	23.89 ± 0.39 μl/ml for MCF-7	Notable cytotoxic impact on the cell lines HEPG2 and MCF-7	^119^
			13.86 ± 0.95 μl/ml for HEP G2		

### 4.2 Anti-angiogenesis activity

Angiogenesis produces new blood vessels from old ones. The initial angiogenesis hypothesis, introduced 40 years ago, stated that tumor development relied on blood vessels for waste, nutrition, oxygen, and spreading to other tissues.^120^ Also advised shutting off tumor blood flow as a treatment, Cancer and other diseases caused by aberrant angiogenesis may be treated by targeting tumor-development-related factors, which are necessary for ovulation, wound healing, and embryonic development.^121^ Although healthy angiogenesis is tightly regulated, abnormalities have been linked to obesity and cancer, resulting in excessive blood vessel development.^103^^,^^104^ Due to their ability to affect cellular processes and interact with biomolecules, silver nanoparticles have been studied for angiogenesis treatment.^122^ Silver nanoparticles alter cell proliferation, migration, angiogenic factors, and anti-inflammatory and anti-microbial actions during angiogenesis.^123^ Silver nanoparticles may boost endothelial cell proliferation, which is necessary for blood vessel formation.^124^ Silver nanoparticles promote endothelial cell growth to generate new blood vessels. Silver nanoparticles may also help endothelial cells migrate to new blood vessel sites.^125^ The start and development of angiogenesis need migration.^126^ Malignant angiogenesis is usually harmful, resulting in aberrant and abundant blood arteries entering tumors.^103^^,^^104^ Research into advanced technologies produced nanotechnology-based medications.^110^, ^111^, ^112^, ^113^^,^^127^ Silver nanoparticles affect VEGF (Vascular endothelial growth factor) and FGF (Fibroblast growth factors) synthesis and activity.^128^ This alteration increases angiogenesis signaling pathways, which stimulates blood vessel growth.^129^ Silver nanoparticles are antimicrobial and anti-inflammatory,^130^ It may facilitate the development of new blood vessels (angiogenesis) by reducing inflammation and preventing infections at the location where the blood vessels are forming.^131^ Nanoparticles have the potential to serve as effective agents for inhibiting the growth of blood vessels or delivering medicine in the context of medical therapy.^132^ In addition, nanoparticles may be directed to certain tissues using various ligands, microRNAs, peptides, and antibodies.^133^ Silver nanoparticles show promise in the treatment of angiogenesis by enhancing the proliferation and migration of endothelial cells, controlling the expression of angiogenic factors, and facilitating the construction of a conducive environment for the formation of new blood vessels.^134^

**Fig. 3:**
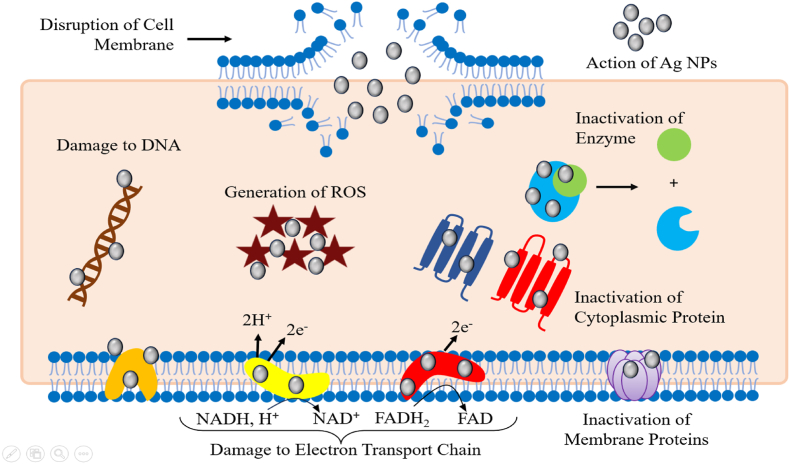
Provides a description. The role of silver nanoparticles as an anti-angiogenic agent.

Napoleone Ferrara is credited with the discovery of vascular endothelial growth factor [VEGF], a well-recognized activator of angiogenesis.^135^ During the process of tumor development and metastasis, there is an excessive expression of VEGF.^136^ The primary transcription of note is the NF-kB element, Nuclear factor - kappa β cells which enhances the production of VEGF.^137^ VEGF enhances the synthesis of A1 and Bcl-2,^138^ indicating the activation of Bcl-2, which is an anti-apoptotic protein.^139^ Anti-angiogenesis techniques are used to halt cancer growth and limit blood vessel formation, preventing tumor growth and progression.^140^ Anti-angiogenesis strategies effectively halted tumor development, ensuring that the tumors did not exceed a size of 1–2 mm^3^ before succumbing to hypoxia.^141^ Methods to reduce angiogenesis include signal transduction blockade, matrix metalloproteinase [MMP] inhibitors, and monoclonal antibodies targeting size, Chemotherapy and antiangiogenic medicines enhance cancer prognosis.^142^ Nevertheless, angiogenesis inhibitors are associated with drawbacks such as reduced sensitivity to radiation, inhibiting VEGF-dependent angiogenesis, and the development of resistance to treatment,^143^ and monotherapy may not be useful in preventing angiogenesis.^144^ The use of a drug carrier led to a substantial enhancement in the biocompatibility and effectiveness of the drugs, namely the angiogenesis inhibitors.^145^ Silver nanoparticles have shown anti-angiogenic properties, which may help combat conditions such as obesity and cancer.^146^ Fig. 3 illustrates the mechanism by which silver nanoparticles function as an anti-angiogenic agent. AgNPs exhibit antiangiogenic properties by downregulating the PI3K/Akt pathway the green synthesis of AgNPs, which is a simple and cost-effective process, has been reported by Baharara et al. successfully created AgNPs using an extract from *Saliva officinalis*, study demonstrated that green AgNPs have a dose-dependent antiangiogenic effect on embryos.^109^ According to research conducted by Yang et al. AgNPs demonstrate antiangiogenic actions by effectively reducing HIF-1 expression levels in a dose-dependent manner AgNPs, administered at a concentration of 100 μg/ml, have the potential to inhibit HIF-1 transcription in MCF-7 cells and, AgNPs at a concentration of 50 μg/ml may effectively suppress HIF-1 expression,^147^ The regulation of GLUT-1 may affect the expression of several targets, such as VEGF-A and glucose transporter type 1, It has been suggested that this modulation might influence the energy sources of tumor cells.,^148^ Table 3 describes several angiogenic agents and strategies.

**Table 3:** Anti-angiogenic drugs and their Mechanism of action.

S·NO	Drugs	Angiogenesis Inhibitory mechanism	References
1	Celecoxib, Rofecoxib	COX-2 inhibitors	^145^,^146^
2	Endostatin	The Inhibition of endothelial-cell survival	^149^
3	Erlotinib, Gefitinib	Inhibitors of the EGFR	^150^
4	Semaxanib, Sunitinib, Sorafenib, Vatalanib	Inhibition of the receptor tyrosine kinase	148, 151, 152
5	Bevacizumab	Target VEGF and inhibit VEGF complexes such as VEGF-A and VEGF-2	^153^
6	GEM 220	Inhibition of the VEGF	^149^,^154^
7	Aflibercept	Targets VEGF and PlGF (Placenta Growth Factor)	^155^
8	Apatinib	Targets VEGFR-2 (Vascular Endothelial Growth Factor Receptor 2)	^156^

### 4.3 Antidiabetic activity

Diabetes is a persistent medical condition characterized by elevated levels of sugar in the blood due to insufficient production of insulin or ineffective absorption of insulin by cells.^157^ The worldwide prevalence of diabetes increases the likelihood of developing heart disease, chronic kidney illness, stroke, leg ulcers, and eye impairment.^158^ Numerous factors contribute to the onset of diabetes, with the primary one being oxidative stress caused by reactive oxygen species [ROS] excessive accumulation of glucose and fatty acids leads to the production of reactive oxygen species (ROS).^159^ The prevalence of frequency in the adult population worldwide is considered to be high, with a rate of 4.7 % in 2014 and a rise of 8.8 % in 2017 it is projected to reach an anticipated rate of 9.9 % by 2045.^160^ The prevalence of diabetes in Southeast Asia is projected to increase by 84 % between 2017 and 2045, with an estimated rise from 82 million persons to 151 million individuals.^161^ Research indicates that oxidative damage may lead to the loss of pancreatic cells, which in turn can cause diabetes difficulties. Hyperglycaemia, conversely, causes diabetic complications due to an imbalance in reactive oxygen species (ROS), resulting in heightened oxidative stress and premature cell death. Research has shown that Diabetes may be effectively managed by lowering the formation of Reactive Oxygen Species (ROS).^162^ An advanced approach is required to address the degenerative nature, which involves the creation of novel pharmaceuticals.^163^ Studies are now progressing on creating a variety of pharmaceuticals to treat diabetes and manage its symptoms. However, the effectiveness of many of these drugs is limited due to their inadequate pharmacokinetic properties.^164^ Nanotechnology is now generating significant attention due to its diverse range of possible applications in several fields, such as antimicrobials and biomaterial manufacturing.^165^ Recent research has focused on exploring the potential of nanomedicine as a therapy for diabetes.^166^ Studies have explored the application of silver nanoparticles in nanomedicine, particularly for addressing diabetes.^167^

The unique physicochemical and biological action of silver nanoparticles has shown potential antidiabetic effects.^168^ Research indicates that silver nanoparticles may possess antidiabetic properties by improving the body's response to insulin, reducing oxidative stress, and increasing cellular glucose uptake.^169^ Studies have shown the efficacy of silver nanoparticles in enhancing insulin sensitivity and regulating blood sugar levels.^170^ Silver nanoparticles possess antioxidant characteristics that may effectively reduce oxidative stress in the human body.^171^ Oxidative stress is intimately linked to diabetes and its repercussions.^172^ Research indicates that silver nanoparticles have the potential to enhance cellular glucose levels, therefore aiding in the regulation of blood sugar.^173^ Diabetes controls the activity of many enzymes, such as α-glucosidase, responsible for converting disaccharides into monosaccharides, and α-amylase, which turns complex carbohydrates into disaccharides consequently, inhibiting these enzymes might be an effective technique for treating diabetes. AgNPs produced in vitro from *Calophyllum tomentosum*,^174^ *Punica granatum*,^175^ *Ficus palmata*,^176^ and *Lonicera japonica*^177^ The AgNPs displayed considerable α-amylase inhibition [AAI] and α-glucosidase inhibition [AGI], indicating that they may have notable anti-diabetic effects. AgNPs synthesized using green methods may exhibit notable anti-diabetic properties due to their elevated levels of AAI (*anti*-α-amylase activity) and AGI (*anti*-glycation activity).^178^ The anti-diabetic effectiveness of these AgNPs was evaluated based on the decrease in blood glucose levels and their impact on other blood physiological processes, such as triglyceride and cholesterol levels.^21^ In addition, the application of *Eysenhardtia polystachya* silver nanoparticles (AgNPs) resulted in enhanced insulin production, decreased hypolipidemia, and provided protection to pancreatic β cells by restarting H_2_O_2_ -induced insulin production in INS-1 cells against oxidative stress.^179^ A zebrafish with diabetes that was exposed to glucose had the same outcomes. Similarly, it was shown that in rats with diabetes caused by streptozotocin, Cassia auriculata AgNPs decreased blood glucose levels and restored other related markers to their normal state.^160^^,^^161^ The presence of silver nanoparticles led to a considerable decrease in the concentrations of glucose and liver enzymes, including ALT, AST, and ALP, the antioxidative activity of silver nanoparticles (AgNPs) indicates the consistent functioning of catalase (CAT) and superoxide dismutase (SOD) enzymes, the histopathological tests of the liver and kidney revealed that the injection of Ag NPs reduced cell damage induced by STZ, demonstrating an antioxidant impact on these vital organs.^180^

**Fig. 4:**
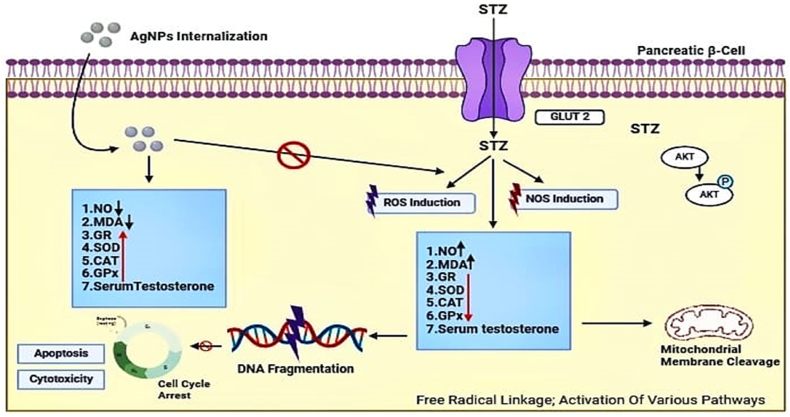
Fig. 4 illustrates the anti-diabetic mechanism of silver nanoparticles.

AgNPs administration in STZ-induced diabetic mice resulted in elevated insulin levels, reduced blood glucose levels, enhanced glucokinase expression, and improved expression of insulin receptor isoform A and GLUT-2.50, Silver nanoparticles (AgNPs) derived from plant extracts are presently extensively used in the field of medicine, namely for targeted medication administration.^181^ The study revealed that diabetic fish had higher blood insulin levels compared to the control zebrafish, suggesting a dysfunction in pancreatic β cells, a concentration of 100 μg/mL of EP [*Eisenhardt polystachya*]/AgNPs resulted in a 54 % decrease in plasma insulin levels compared to the group of diabetic fish produced by glucose, which is similar to the zebrafish group with normal blood sugar levels.^182^ Increased glucose levels promote the synthesis of insulin, but continuous exposure may impair the ability of insulin to be secreted and reduce its long-term viability^183^ the findings indicate that EP/AgNPs can enhance both cell survival and insulin production in zebrafish when exposed to elevated glucose levels, the study demonstrated the benefits of using EP/AgNPs for treating glucose-induced diabetes in zebrafish, which aligns with earlier research.^184^ AgNPs produced by biological means improve the survival of pancreatic β-cells and increase insulin production in zebrafish with diabetes caused by glucose AgNPs have the potential to initiate the synthesis of insulin in INS-1 cells that have been stimulated by hydrogen peroxide this effect may be attributed to the ability of AgNPs to safeguard the cells from oxidative harm and potential to function as therapeutic nanoparticles for the prevention of diabetes.^185^ Fig. 4 illustrates the anti-diabetic characteristic of silver nanoparticles. The research emphasizes that AgNPs have antioxidant characteristics, which enable them to eliminate free radicals and alleviate oxidative stress. Diabetes is regulated by oxidative stress, which leads to the destruction of pancreatic β-cells and the development of insulin resistance in peripheral organs.^186^ Silver nanoparticles (AgNPs) have been shown to possess anti-inflammatory properties by inhibiting the production of pro-inflammatory cytokines and preventing the activation of inflammatory signaling pathways.^187^ AgNPs enhance insulin sensitivity and glucose metabolism by reducing inflammation.^188^ The mechanisms that increase insulin production include phosphoinositide 3-kinase [PI3K] and cAMP-dependent protein kinase [PKA].^189^ The multiple routes of AgNPs contribute to their anti-diabetic effects by targeting oxidative stress, inflammation, and insulin production However, further research is necessary to fully conclude the specific biochemical mechanisms that support the anti-diabetic effects of AgNPs and their potential therapeutic significance in the treatment of diabetes.

### 4.4 Wound healing

The skin, the largest organ in the body, plays a vital role in maintaining homeostasis, regulating body temperature, and safeguarding against poisons, infections, and injuries.^190^ There are two sorts of wounds: acute wounds and chronic wounds.^191^ Persistent ulcers are characterized by their resistance to healing and their failure to achieve anatomical or functional integrity.^192^ A chronic wound is characterized as a lesion or ulcer that is challenging to cure and remains unhealed for a period of three months or more.^193^ Wound healing involves many cellular pathways that occur in overlapping stages, including the inflammatory phase, the proliferative phase, and the remodeling phase these phases work together to repair and restore the integrity and functioning of the tissue.^194^ During an inflammatory response in the blood vessels, injured blood vessels undergo contraction, leading to the clustering of thrombocytes inside a fibrin network, resulting in the process of coagulation.^195^ Various growth factors and cytokines released at the site of the wound have a substantial impact on the process of wound healing,^196^ Due to the complexity of the procedure, several variables might inhibit, delay, or increase patient morbidity and mortality, and result in unsatisfactory cosmetic results, as well as significant discomfort.^197^ Chronic wounds are prevalent among the elderly population, significantly diminishing their quality of life.^198^ Chronic wounds are often known as the "Silent Epidemic" because to their significant influence on patients' physical and mental well-being.^199^ Approximately 6 million persons in the United States are affected with chronic ulcers, with a higher prevalence among the elderly and diabetics.^200^ Diabetes impacts a total of 20 million people in the United States, and it is projected that the number of cases will increase fourfold to reach 4444 by the year 2030, Diabetic foot ulcers were present in 15 % of these people, and they are strongly associated with the majority of amputations.^197^ Underlying conditions such as diabetes and neuropathy may also affect the composition of wound fluid, which can vary significantly due to changes in the microenvironment and tissue restructuring throughout the healing process. The biochemical processes involved in wound healing are categorized into several stages, including the inflammatory response, cellular proliferation, synthesis of extracellular matrix components, and tissue remodeling.^201^ The use of silver in wound treatment has a long history and is considered a conventional therapeutic approach.^202^ Commercially available silver-based ointments and biomedical products containing silver nanoparticles (AgNP) are now accessible for many medicinal purposes owing to their wide-ranging antibacterial properties.^203^ In recent years, there has been substantial growth in the use of silver nanoparticles and biopolymers for wound therapy.^204^

**Fig. 5:**
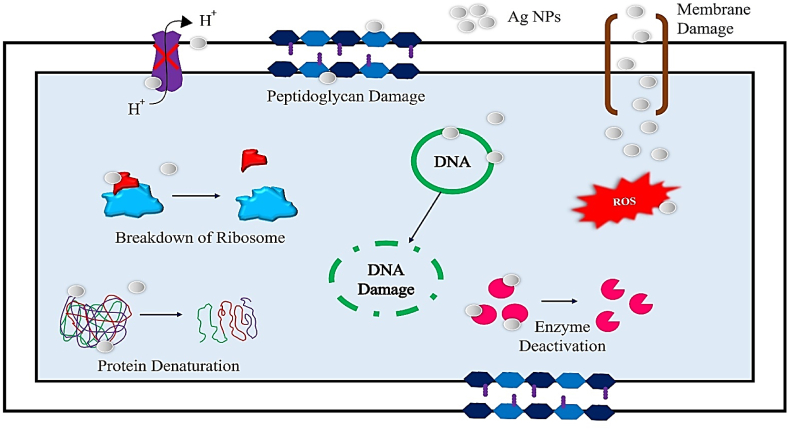
Illustrates the intricate mechanism of silver nanoparticles in the process of wound healing.

Dai et al. assessed the wound-healing properties of the composite of antimicrobial peptide-AgNPs in vivo by using a diabetic rat model, the research demonstrated enhanced wound healing without any adverse effects on dermal tissues, as well as reinforced interactions between peptide and lipopolysaccharide components on bacteria, suggesting a wide-ranging effectiveness without promoting bacterial resistance.^205^ Kumar et al. conducted a study to examine the healing effects of silver nanoparticles (AgNPs) on wounds in albino rat models. The researchers observed that rats treated with cream formulations containing different concentrations of AgNPs had reduced wound area, increased collagen deposition, decreased presence of macrophages and tissue swelling, and higher numbers of fibroblasts, particularly at the highest concentrations of silver.^203^ Bandages coated with silver nanoparticles [14 nm] effectively decreased inflammation and scarring, inhibited bacterial growth, and facilitated healing in BALB/C mice that had suffered a thermal injury.^206^ Silver nanoparticles are often used to promote wound healing while preventing infection, either alone or in combination with antibacterial drugs.^207^ A study has shown that dressings containing silver nanoparticles do not affect the growth of fibroblasts and keratinocytes, which are responsible for promoting the restoration of normal skin growth, in patients with partial-thickness burns and fibroblast cell culture in vitro.^208^ In addition, the combination of silver nanoparticles with medicines exhibits greater efficacy against bacterial infections. Furthermore, these findings indicate the possible use of silver nanoparticles in combination with antibacterial drugs for the management of skin wound infections.^209^ Rujitanaroj et al.^210^ reported a randomized controlled trial that included community nursing clients who had leg ulcers affected by bacterial load these patients received either silver or iodine treatments for their wounds the findings demonstrated that the use of silver nanoparticle compounds in patients' treatment resulted in accelerated recuperation.^211^ Silver nanoparticles hold promise for enhancing various products utilized in wound healing, such as bandages, gauzes, sutures, plasters, and other lotions and ointments.^212^ During the process of wound healing, thrombocytes and immune cells gather at the site of the injury in response to both the damaged extracellular matrix (ECM) and the cells present in the tissue.^213^ Platelets are the first cells to enter, aiding in the coagulation cascade to prevent further blood loss and creating a temporary extracellular matrix for future cell infiltration.^214^ Platelets release TGF-β1 and platelet-derived growth factors [PDGFs], which stimulate fibroblasts and mesenchymal cells and attract neutrophils and macrophages.^215^ Platelet abnormalities are often associated with impaired wound healing, and the use of autologous PRP improves wound resolution.^216^ Silver nanoparticles (AgNPs) enhance the activity of fibroblasts, the key cellular components responsible for the synthesis of collagen, resulting in increased collagen production collagen is an essential constituent of the extracellular matrix (ECM), which plays a crucial role in providing structural reinforcement to many tissues, Silver nanoparticles (AgNPs) enhance the structural integrity and robustness of the extracellular matrix (ECM) by promoting the accumulation of collagen, hence facilitating accelerated healing of wounds and regeneration of tissues.^217^ Silver nanoparticles (AgNPs) modulate the activity of matrix metalloproteinases (MMPs), which are enzymes responsible for extracellular matrix (ECM) remodeling MMPs play a crucial role in the degradation and remodeling of the extracellular matrix (ECM) throughout various phases of the wound healing process, Silver nanoparticles (AgNPs) aid in regulating the activity of mitochondrial membrane potential (MMP), ensuring appropriate reconstruction of the extracellular matrix (ECM) without excessive degradation or buildup of its constituents.^218^ Silver nanoparticles (AgNPs) have antioxidant characteristics that help eliminate free radicals and alleviate oxidative stress in the wound microenvironment, Oxidative stress has the potential to harm the components of the extracellular matrix (ECM) and impair its function, leading to a delay in the healing process of wounds, Silver nanoparticles (AgNPs) help maintain the integrity and efficacy of the extracellular matrix (ECM) by minimizing oxidative damage, hence promoting wound healing.^219^ The mechanism by which silver nanoparticles contribute to the process of wound healing is explained in Fig. 5. In brief, the ability of AgNPs to enhance the integrity, organization, and remodeling of the extracellular matrix contributes to their usefulness in facilitating the healing of wounds and regeneration of tissues. The aforementioned attributes render AgNPs valuable therapeutic agents for wound care applications.

### 4.5 Antimicrobial application of AgNPs

Silver has notable antibacterial qualities, making it a valuable remedy for a range of infection management therapies.^220^ Nanotechnology has revolutionized the field of antimicrobials by offering novel approaches to combat microbial diseases.^221^ Silver nanoparticles (AgNPs) have shown high efficacy against a wide range of pathogens, including over 650 types of bacteria, fungi, and viruses.^222^ Biologically synthesized AgNPs have shown characteristics that make them suitable as alternative antibacterial agents in combating multidrug resistance, after years of research.^223^ Silver nanoparticles possess significant antibacterial properties. Studies have shown that silver nanostructures are especially efficient in inhibiting the proliferation of bacteria and other pathogens.^224^ Silver nanoparticles provide potent and extensive coverage over a wide range of wavelengths.^225^ Research has shown that Gram-negative bacteria are more vulnerable to silver nanoparticles compared to Gram-positive bacteria examples include *Escherichia coli, Pseudomonas aeruginosa*, and methicillin-resistant *Staphylococcus aureus* (MRSA),^226^ the variation in sensitivity might be ascribed to disparities in the structural characteristics of the two bacterial species, together with the dimensions and configuration of the silver nanoparticles. Moreover, it was shown that the inclusion of silver nanoparticles enhanced the antibacterial efficacy of many types of antibiotics.^227^

Nanda and Saravanan conducted a study to examine the antibacterial efficacy of silver nanoparticles against various bacterial species. The most potent antibacterial effect was shown against methicillin-resistant *Staphylococcus aureus*.^228^ Silver nanoparticles (AgNPs) have potent antibacterial capabilities, yet, the specific processes behind this effect remain unclear. Various concepts have been documented about the antibacterial characteristics of AgNPs, such as the degradation of the bacterial membrane and the subsequent release of cellular contents,^229^ producing reactive oxygen species [ROS],^230^ inhibiting respiratory chains,^231^ destroying DNA structure, preventing DNA replication^232^ and deactivating enzymes and denaturing proteins.^233^ Silver nanoparticles (AgNPs) demonstrate substantial and wide-ranging antibacterial activity due to these mechanisms. AgNPs have the potential to replace advanced biomedical treatments, such as bone, wound, and catheter repair.^6^ Kim et al. conducted a study to examine the antibacterial properties and mechanism of action of silver nanoparticles against *Escherichia coli* they produced the silver nanoparticles using a chemical reduction method and analyzed their physical and chemical properties, as well as their antibacterial effects the findings demonstrated that the silver nanoparticles were highly potent, with a minimum inhibitory concentration (MIC) of 0.1 mg/L.^234^ The scientists used transmission electron microscopy (TEM) and scanning electron microscopy (SEM) as well.^235^ To investigate the interaction between silver nanoparticles and bacterial cells, researchers observed that the nanoparticles attached to the bacterial cell membrane, resulted in structural harm and ultimately caused cell demise.^236^ Moreover, the silver nanoparticles caused harm to the bacterial cell membrane, leading to the release of cellular contents and finally to the death of the cell.^237^ Current work offers valuable insights into the efficacy of silver nanoparticles as antibacterial agents like, *Escherichia coli*.^238^ Rai et al. researched to investigate the antibacterial effects of silver nanoparticles on drug-resistant bacterial strains^239^ synthesized silver nanoparticles and assessed their efficacy against several medically important bacteria, such as methicillin-resistant *Staphylococcus aureus* [MRSA] and vancomycin-resistant Enterococcus [VRE].^240^ The nanoparticles exhibited exceptional antibacterial efficacy against all examined pathogens, with minimum inhibitory concentrations (MICs) ranging from 1 to 8 μg/mL.^241^ Furthermore, the nanoparticles were seen to impede the growth of biofilms and eradicate existing biofilms, suggesting their potential as a therapeutic choice for diseases associated with biofilms.^242^ The research also investigated the mechanism by which silver nanoparticles exert their effects, revealing that they induce oxidative stress and DNA damage in bacterial cells, ultimately resulting in cell death. The results indicate that silver nanoparticles have the potential to be a practical substitute for conventional antibiotics in combating drug-resistant bacterial diseases.^243^

Silver nanoparticles (AgNPs) demonstrate potent antiviral activity against the influenza A [H1N1] virus,^244^ the herpes simplex virus [HSV],^245^ the human parainfluenza virus [HPIV],^246^ and the hepatitis B virus [HBV].^247^ AgNPs with a size less than 10 nm have significant antiviral efficacy.^139^^,^^241^ This may be due to their potent adhesion to the surface of the virus and their extensive reaction area.^248^ Silver nanoparticles (AgNP) have two unique modes of interaction with dangerous viruses it binds to the viral envelope, inhibiting its ability to bind to cellular receptors,^249^ it binds to the DNA or RNA of the virus, inhibiting its replication and dissemination inside the host cells.^250^ Gaining insight into the mechanisms by which nanoparticles combat various kinds of viruses might facilitate the creation of innovative antiviral treatments. The antiviral properties of silver nanoparticles might potentially prevent or mitigate the risk of infection from an epidemic viral sickness like COVID-19.^23^ AgNPs might be used as a therapeutic approach in response to the ongoing global COVID-19 pandemic, which has led to more than 8 million confirmed cases.^251^ Currently, there is a study expressing an opinion on the use of silver nanoparticles as an antiviral medicine for COVID-19 patients, highlighting its little potential for causing negative effects.^252^ The antiviral properties of AgNP may be beneficial for several industries, particularly healthcare since they can effectively combat general infection challenges.^253^ Elechiguerra et al. examined the antiviral properties of silver nanoparticles against HIV-1, the pathogen responsible for AIDS.^254^ The synthesis of silver nanoparticles was conducted, followed by in vitro testing to evaluate their antiviral effectiveness against HIV-1 the findings indicate that the silver nanoparticles effectively inhibit viral reproduction by attaching to the viral envelope, namely the glycoprotein gp120^255^ Inhibiting its interaction with the CD4 receptor on host cells. Current work provides evidence that silver nanoparticles might serve as a promising novel antiviral treatment for HIV-1. Speshock et al. conducted a study to examine the antiviral properties of silver nanoparticles against respiratory syncytial virus [RSV], a prevalent respiratory disease the study included the production of silver nanoparticles and their assessment for effectiveness against RSV. The results showed that the nanoparticles effectively hindered the reproduction of the virus by interacting with the glycoproteins on its outer membrane,^256^ Specifically, the fusion protein is crucial for the virus to successfully penetrate host cells.^257^ This interaction impeded the merging of the viral envelope with the membrane of the host cell, hence inhibiting viral entry and replication.^258^ In addition, there is a growing need for more potent antifungal medications to treat fungal infections, since these illnesses are becoming more prevalent and drug-resistant forms of fungi have emerged as a result. Silver nanoparticles (AgNPs) have garnered significant attention due to their exceptional antibacterial properties, which also render them potentially effective as antifungal drugs.^252^^,^^253^

**Fig. 6:**
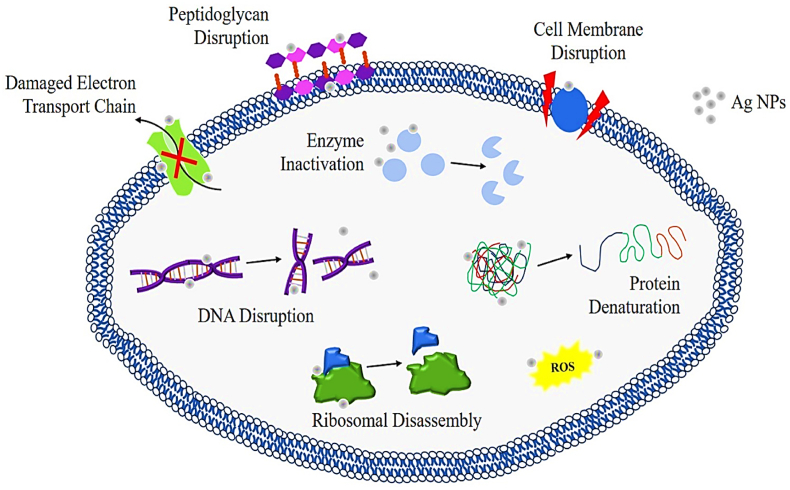
Indicates the antimicrobial properties of silver nanoparticles.

The research found that the combination of fluconazole and silver nanoparticles had the most powerful inhibitory effect on *Candida albicans*.^259^ The fungus Alternaria alternative was used to produce silver nanoparticles externally,^47^ The study found that the presence of silver nanoparticles at concentrations between 30 and 200 mg/L significantly suppressed the development of the fungus.^260^ According to Jalal et al.'s TEM investigation,^261^ Exposing Candida cells to silver nanoparticles resulted in significant distortion of the cells.^262^ In addition, the interaction between the silver nanoparticles and the cell wall and membrane worsened the constriction of the fungus.^263^ The cell membrane was disturbed, resulting in damage and loss of membrane integrity, which inhibits the usual budding process.^264^ Jalal et al. further illustrated the antibacterial attributes of silver nanoparticles against Candida species they affirmed that these nanoparticles can restrict the proliferation and development of germ tubes and biofilms, as well as the production of enzymes by Candida species.^265^ Silva et al. reported that silver nanoparticles [AgNPs] have antifungal activities against *Candida albicans* and *Trichophyton rubrum* in a mouse model of cutaneous infection.^266^ AgNPs were synthesized using a green method and analyzed using various techniques the antifungal activity of AgNPs was assessed in a mouse model of skin infection, the mechanism of action was investigated through histological analysis and immunohistochemistry, the study showed that AgNPs reduced the production of inflammatory cytokines and chemokines in the infected skin tissue the findings indicate that AgNPs effectively reduced the fungal load and inflammation in the diseased skin tissue, demonstrating their strong antifungal action against *Candida albicans* and *Trichophyton rubrum*.^261^, ^262^, ^263^ Li et al. examined the fungicidal properties of silver nanoparticles (AgNPs) against *Aspergillus fumigatus*^139^ the synthesis of AgNPs was carried out using a chemical reduction approach, and the nanoparticles were thoroughly characterized^267^ the effectiveness of AgNPs in inhibiting fungal growth was evaluated using the broth microdilution technique,^268^ While the method by which they operate was examined using transmission electron microscopy and scanning electron microscopy.^269^ The findings were remarkable, indicating that AgNPs exhibited potent antifungal properties against *Aspergillus fumigatus*, with a minimum inhibitory concentration [MIC] of 0.2 mg/L.^270^ The TEM and SEM investigations revealed that the AgNPs interacted with the fungal cell membrane, causing its breakdown and the subsequent release of cellular contents.^271^ In addition, AgNPs stimulated the generation of reactive oxygen species [ROS] in *Aspergillus fumigatus*, resulting in oxidative stress and subsequent cell demise. This mechanism not only elucidates the antifungal mechanism of AgNPs but also explains their potential as powerful antifungal agents against *Aspergillus fumigatus*.^272^ Overall, silver nanoparticles have a wide range of antifungal properties, act on several targets, and have a low likelihood of developing resistance, making them a very useful antimicrobial agent for various medicinal uses. Fig. 6 depicts the antimicrobial properties of silver nanoparticles. The current study intends to investigate the possibility of employing silver nanoparticles in formulations to lessen infectious illnesses and antibiotic resistance while also improving their efficacy.

## 5 Conclusion and future directions

This review focuses on silver nanoparticles and their characterization, specifically examining several techniques of synthesis and the ecologically sustainable and cost-efficient biological approach. The present work highlights the varied therapeutic capabilities of plant-derived AgNPs, like cancer, anti-angiogenesis, diabetes, wound healing, and antimicrobial activity. Although the mechanisms of action and therapeutic potential are recognized, there is still a significant gap in its application in healthcare, to bridge this gap, it is necessary to conduct thorough research on more efficient biomolecules for reducing silver ions and generating stable silver nanoparticles for therapeutical use, and these improvements hold great promise for improving therapies in human health and medicine by utilizing natural extracts. Despite the absence of clear mechanisms, the accumulation of silver nanoparticles can lead to the development of argyria, and also the excessive ingestion of silver nanoparticles can potentially cause damage to the kidneys and neurological system. The novelty of the current investigation reports the unique intrinsic activities of bioinspired AgNPs in both, in-vivo and vivo studies in combination with phytochemicals, and also highlights further research, including both animal and human investigations, is required to report a significant therapeutic window, safety, and efficiency of silver nanoparticles, and to resolve stability and bioavailability concerns. From the viewpoint of future research targeted delivery techniques, cellular uptake, distribution, routes of clearance, and molecular interactions involving AgNPs are required that hold promise for future advancements. The antibacterial and wound-healing capabilities of AgNPs make them suitable for incorporating advanced biomaterials and tissue engineering structures, enabling the development of innovative approaches for tissue repair and regeneration. Silver nanoparticles (AgNPs) possess optical and electrochemical features that may make them well-suited for use in biosensors and diagnostic tools. These properties may enable early illness diagnosis and monitoring in the future. The integration of nanotechnology and biology is poised to revolutionize medicine with AgNPs at the forefront transforming treatments, diagnostics, and pharmaceutical delivery to improve healthcare outcomes.

## Ethical approval

The author does not have any ethical issues.

## Funding

The authors have no relevant financial or non-financial interests to disclose.

## CRediT authorship contribution statement

Karthik K Karunakar: Investigation, Formal analysis, Writing – original draft. Binoy Varghese Cheriyan Conceptualization, Writing – original draft, Supervision, Writing – review & editing. Krithikeshvaran: data collection, writing draft, Gnanisha: data collection, writing draft, Abinavi B; Writing – original draft.

## Declaration of competing interest

Author do not have any Conflict of Interest.

## References

[1] Almatroudi A. (2020). Silver nanoparticles: synthesis, characterisation and biomedical applications. Open Life Sci.

[2] Najahi-Missaoui W., Arnold R.D., Cummings B.S. (2021). Safe nanoparticles: are we there yet?. Int J Mol Sci.

[3] Missaoui W.N., Arnold R.D., Cummings B.S. (2018). Toxicological status of nanoparticles: what we know and what we don't know. Chem Biol Interact.

[4] Malik M., Aamir Iqbal M., Iqbal Y. (2022). Biosynthesis of silver nanoparticles for biomedical applications: a mini review. Inorg Chem Commun.

[5] Shanmuganathan R., Karuppusamy I., Saravanan M. (2019). Synthesis of silver nanoparticles and their biomedical applications - a comprehensive review. Curr Pharmaceut Des.

[6] Xu L., Wang Y.Y., Huang J., Chen C.Y., Wang Z.X., Xie H. (2020). Silver nanoparticles: synthesis, medical applications and biosafety. Theranostics.

[7] Iravani S., Korbekandi H., Mirmohammadi S.V., Zolfaghari B. (2014). Synthesis of silver nanoparticles: chemical, physical and biological methods. Research in Pharmaceutical Sciences.

[8] Zhang X.F., Liu Z.G., Shen W., Gurunathan S. (2016). Silver nanoparticles: synthesis, characterization, properties, applications, and therapeutic approaches. Int J Mol Sci.

[9] Pantidos N. (2014). Biological synthesis of metallic nanoparticles by bacteria, fungi and plants. J Nanomed Nanotechnol.

[10] Habeeb Rahuman H.B., Dhandapani R., Narayanan S. (2022). Medicinal plants mediated the green synthesis of silver nanoparticles and their biomedical applications. IET Nanobiotechnol.

[11] Bhuyar P., Rahim M.H.A., Sundararaju S., Ramaraj R., Maniam G.P., Govindan N. (2020). Synthesis of silver nanoparticles using marine macroalgae Padina sp. and its antibacterial activity towards pathogenic bacteria. Beni-Suef Univ J Basic Appl Sci.

[12] Fernandez C.C., Sokolonski A.R., Fonseca M.S. (2021). Applications of silver nanoparticles in dentistry: advances and technological innovation. Int J Mol Sci.

[13] Yousaf H., Mehmood A., Ahmad K.S., Raffi M. (2020). Green synthesis of silver nanoparticles and their applications as an alternative antibacterial and antioxidant agents. Mater Sci Eng C.

[14] Porenczuk A., Grzeczkowicz A., Maciejewska I. (2019). An initial evaluation of cytotoxicity, genotoxicity and antibacterial effectiveness of a disinfection liquid containing silver nanoparticles alone and combined with a glass-ionomer cement and dentin bonding systems. Adv Clin Exp Med.

[15] Gurunathan S., Han J.W., Kim E.S., Park J.H., Kim J.H. (2015). Reduction of graphene oxide by resveratrol: a novel and simple biological method for the synthesis of an effective anticancer nanotherapeutic molecule. Int J Nanomed.

[16] Prabhu S., Poulose E.K. (2012). Silver nanoparticles: mechanism of antimicrobial action, synthesis, medical applications, and toxicity effects. Int Nano Lett.

[17] Flores-López L.Z., Espinoza-Gómez H., Somanathan R. (2019). Silver nanoparticles: electron transfer, reactive oxygen species, oxidative stress, beneficial and toxicological effects. J Appl Toxicol.

[18] Wang Z.X., Chen C.Y., Wang Y. (2019). Ångstrom-scale silver particles as a promising agent for low-toxicity broad-spectrum potent anticancer therapy. Adv Funct Mater.

[19] Rai M., Birla S., Ingle A.P. (2014). Nanosilver: an inorganic nanoparticle with myriad potential applications. Nanotechnol Rev.

[20] Doyle-Delgado K., Chamberlain J.J., Shubrook J.H., Skolnik N., Trujillo J. (2020). Pharmacologic approaches to glycemic treatment of type 2 diabetes: synopsis of the 2020 American diabetes association's standards of medical care in diabetes clinical guideline. Ann Intern Med.

[21] Sengottaiyan A., Aravinthan A., Sudhakar C. (2016). Synthesis and characterization of Solanum nigrum-mediated silver nanoparticles and its protective effect on alloxan-induced diabetic rats. J Nanostructure Chem..

[22] Sim W., Barnard R.T., Blaskovich M.A.T., Ziora Z.M. (2018). Antimicrobial silver in medicinal and consumer applications: a patent review of the past decade (2007–2017). Antibiotics.

[23] Salleh A., Naomi R., Utami N.D. (2020). The potential of silver nanoparticles for antiviral and antibacterial applications: a mechanism of action. Nanomaterials.

[24] Rai M., Kon K., Ingle A., Duran N., Galdiero S., Galdiero M. (2014). Broad-spectrum bioactivities of silver nanoparticles: the emerging trends and future prospects. Appl Microbiol Biotechnol.

[25] Gunasekaran T., Nigusse T., Dhanaraju M.D. (2011). Silver nanoparticles as real topical bullets for wound healing. Journal of the American College of Clinical Wound Specialists.

[26] Sharad Kamble Mr, Kaveri Bhosale Miss, Mahesh Mohite Mr, Navale Mrs Swapnali (2023). Methods of preparation of nanoparticles. Int J Adv Res Sci Commun Technol..

[27] Prasad Yadav T., Manohar Yadav R., Pratap Singh D. (2012). Mechanical milling: a top down approach for the synthesis of nanomaterials and nanocomposites. Nanosci Nanotechnol.

[28] Kuntyi Kytsya R., Mertsalo I.P., Mazur S. (2019). Electrochemical synthesis of silver nanoparticles by reversible current in solutions of sodium polyacrylate. Colloid Polym Sci.

[29] Lee D.K., Kang Y.S. (2004). Synthesis of silver nanocrystallites by a new thermal decomposition method and their characterization. ETRI J.

[30] Jung J.H., Cheol Oh H., Soo Noh H., Ji J.H., Soo Kim S. (2006). Metal nanoparticle generation using a small ceramic heater with a local heating area. J Aerosol Sci.

[31] Torras M., Roig A. (2020). From silver plates to spherical nanoparticles: snapshots of microwave-assisted polyol synthesis. ACS Omega.

[32] Siegel J., Kvítek O., Ulbrich P., Kolská Z., Slepička P., Švorčík V. (2012). Progressive approach for metal nanoparticle synthesis. Mater Lett.

[33] Kmis F.E., Fissan H., Rellinghaus B. (2000). Sintering and evaporation characteristics of gas-phase synthesis of size-selected PbS nanoparticles. Mater Sci Eng B Solid-State Mater Adv Technol.

[34] Lee S.H., Jun B.H. (2019). Silver nanoparticles: synthesis and application for nanomedicine. Int J Mol Sci.

[35] Shameli K., Ahmad M Bin, Yunus W.M.Z.W., Ibrahim N.A., Gharayebi Y., Sedaghat S. (2010). Synthesis of silver/montmorillonite nanocomposites using γ-irradiation. Int J Nanomed.

[36] Shameli K., Ahmad M Bin, Yunus W.M.Z.W. (2010). Green synthesis of silver/montmorillonite/chitosan bionanocomposites using the UV irradiation method and evaluation of antibacterial activity. Int J Nanomed.

[37] Elsupikhe R.F., Shameli K., Ahmad M.B., Ibrahim N.A., Zainudin N. (2015). Green sonochemical synthesis of silver nanoparticles at varying concentrations of κ-carrageenan. Nanoscale Res Lett.

[38] Fahimirad S., Ajalloueian F., Ghorbanpour M. (2019). Synthesis and therapeutic potential of silver nanomaterials derived from plant extracts. Ecotoxicol Environ Saf.

[39] Gurunathan S., Han J.W., Park J.H., Kim J.H. (2014). A green chemistry approach for synthesizing biocompatible gold nanoparticles. Nanoscale Res Lett.

[40] Gurunathan S., Han J.W., Park J.H. (2015). Reduced graphene oxide-silver nanoparticle nanocomposite: a potential anticancer nanotherapy. Int J Nanomed.

[41] Kalimuthu K., Suresh Babu R., Venkataraman D., Bilal M., Gurunathan S. (2008). Biosynthesis of silver nanocrystals by Bacillus licheniformis. Colloids Surf B Biointerfaces.

[42] Kalishwaralal K., Deepak V., Ram Kumar Pandian S.B. (2010). Biosynthesis of silver and gold nanoparticles using Brevibacterium casei. Colloids Surf B Biointerfaces.

[43] Gurunathan S., Han J.W., Dayem A.A. (2013). Green synthesis of anisotropic silver nanoparticles and its potential cytotoxicity in human breast cancer cells (MCF-7). J Ind Eng Chem.

[44] Gurunathan S., Jeong J.K., Han J.W., Zhang X.F., Park J.H., Kim J.H. (2015). Multidimensional effects of biologically synthesized silver nanoparticles in Helicobacter pylori, Helicobacter felis, and human lung (L132) and lung carcinoma A549 cells. Nanoscale Res Lett.

[45] Gurunathan S. (2015). Biologically synthesized silver nanoparticles enhances antibiotic activity against Gram-negative bacteria. J Ind Eng Chem.

[46] Shankar S., Rhim J.W. (2015). Amino acid mediated synthesis of silver nanoparticles and preparation of antimicrobial agar/silver nanoparticles composite films. Carbohydr Polym.

[47] Gajbhiye M., Kesharwani J., Ingle A., Gade A., Rai M. (2009). Fungus-mediated synthesis of silver nanoparticles and their activity against pathogenic fungi in combination with fluconazole. Nanomed Nanotechnol Biol Med.

[48] Mukherjee P., Ahmad A., Mandal D. (2001). Fungus-mediated synthesis of silver nanoparticles and their immobilization in the mycelial matrix: a novel biological approach to nanoparticle synthesis. Nano Lett.

[49] Rauwel P., Küünal S., Ferdov S., Rauwel E. (2015). A review on the green synthesis of silver nanoparticles and their morphologies studied via TEM. Adv Mater Sci Eng.

[50] Lengke M.F., Fleet M.E., Southam G. (2007). Biosynthesis of silver nanoparticles by filamentous cyanobacteria from a silver(I) nitrate complex. Langmuir.

[51] Marchev A.S., Yordanova Z.P., Georgiev M.I. (2020). Green (cell) factories for advanced production of plant secondary metabolites. Crit Rev Biotechnol.

[52] Rónavári A., Igaz N., Adamecz D.I. (2021). Green silver and gold nanoparticles: biological synthesis approaches and potentials for biomedical applications. Molecules.

[53] Majoumouo M.S., Sibuyi N.R.S., Tincho M.B., Mbekou M., Boyom F.F., Meyer M. (2019). Enhanced anti-bacterial activity of biogenic silver nanoparticles synthesized from Terminalia mantaly extracts. Int J Nanomed.

[54] Simon S., Sibuyi N.R.S., Fadaka A.O., Meyer M., Madiehe A.M., du Preez M.G. (2021). The antimicrobial activity of biogenic silver nanoparticles synthesized from extracts of Red and Green European pear cultivars. Artif Cells, Nanomed Biotechnol.

[55] Tyavambiza C., Elbagory A.M., Madiehe A.M., Meyer M., Meyer S. (2021). The antimicrobial and anti-inflammatory effects of silver nanoparticles synthesised from cotyledon orbiculata aqueous extract. Nanomaterials.

[56] Chand K., Cao D., Eldin Fouad D. (2020). Green synthesis, characterization and photocatalytic application of silver nanoparticles synthesized by various plant extracts. Arab J Chem.

[57] Shirsul J., Tripathi A., Mohanta D., Ankamwar B. (2024). Monstera deliciosa mediated single step biosynthesis of gold nanoparticles by bottom-up approach and its non-antimicrobial properties. 3 Biotech..

[58] Patel V., Berthold D., Puranik P., Gantar M. (2015). Screening of cyanobacteria and microalgae for their ability to synthesize silver nanoparticles with antibacterial activity. Biotechnol Reports.

[59] Oslan S.N.H., Tan J.S., Oslan S.N. (2021). Haematococcus pluvialis as a potential source of astaxanthin with diverse applications in industrial sectors: current research and future directions. Molecules.

[60] Agnihotri S., Mukherji S., Mukherji S. (2014). Size-controlled silver nanoparticles synthesized over the range 5-100 nm using the same protocol and their antibacterial efficacy. RSC Adv.

[61] Ganaie S.U., Abbasi T., Abbasi S.A. (2015). Green synthesis of silver nanoparticles using an otherwise worthless weed mimosa (Mimosa pudica): feasibility and process development toward shape/size control. Part Sci Technol.

[62] Patil R.S., Kokate M.R., Jambhale C.L., Pawar S.M., Han S.H., Kolekar S.S. (2012). One-pot synthesis of PVA-capped silver nanoparticles their characterization and biomedical application. Adv Nat Sci Nanosci Nanotechnol.

[63] Merga G., Wilson R., Lynn G., Milosavljevic B.H., Meisel D. (2007). Redox catalysis on “naked” silver nanoparticles. J Phys Chem C.

[64] Dang T.M.D., Le T.T.T., Fribourg-Blanc E., Dang M.C. (2012). Influence of surfactant on the preparation of silver nanoparticles by polyol method. Adv Nat Sci Nanosci Nanotechnol.

[65] Zhang Q., Li N., Goebl J., Lu Z., Yin Y. (2011). A systematic study of the synthesis of silver nanoplates: is citrate a “magic” reagent?. J Am Chem Soc.

[66] Hosseinpour-Mashkani S.M., Ramezani M. (2014). Silver and silver oxide nanoparticles: synthesis and characterization by thermal decomposition. Mater Lett.

[67] Talebi J., Halladj R., Askari S. (2010). Sonochemical synthesis of silver nanoparticles in Y-zeolite substrate. J Mater Sci.

[68] Ashraf J.M., Ansari M.A., Khan H.M., Alzohairy M.A., Choi I. (2016). Green synthesis of silver nanoparticles and characterization of their inhibitory effects on AGEs formation using biophysical techniques. Sci Rep.

[69] Ivanisevic I. (2010). Physical stability studies of miscible amorphous solid dispersions. J Pharmaceut Sci.

[70] Cabral M., Pedrosa F., Margarido F., Nogueira C.A. (2013). End-of-life Zn-MnO2 batteries: electrode materials characterization. Environ Technol.

[71] Dey A., Mukhopadhyay A.K., Gangadharan S., Sinha M.K., Basu D. (2009). Characterization of microplasma sprayed hydroxyapatite coating. J Therm Spray Technol.

[72] Ananias D., Almeida Paz F.A., Carlos L.D., Rocha J. (2013). Microporous and Mesoporous Materials.

[73] Singh D.K., Pandey D.K., Yadav R.R., Singh D. (2013). A study of ZnO nanoparticles and ZnO-EG nanofluid. J Exp Nanosci.

[74] Warren B.E. (1941). X-ray diffraction methods. J Appl Phys.

[75] Pecharsky V.K., Zavalij P.Y. (2005). Fundamentals of Powder Diffraction and Structural Characterization of Materials.

[76] Dorset D.L. (1998). X-Ray diffraction: a practical approach. Microsc Microanal.

[77] Zscherp C., Barth A. (2001). Reaction-induced infrared difference spectroscopy for the study of protein reaction mechanisms. Biochemistry.

[78] Rohman A., Man Y.B.C. (2010). Fourier transform infrared (FTIR) spectroscopy for analysis of extra virgin olive oil adulterated with palm oil. Food Res Int.

[79] Hall J.B., Dobrovolskaia M.A., Patri A.K., McNeil S.E. (2007). Characterization of nanoparticles for therapeutics. Nanomedicine.

[80] Ratner B.D., Hoffman A.S., Schoen F.J., Lemons J.E. (2004).

[81] Pawley James (1997). The development of field-emission scanning electron microscopy for imaging biological surfaces. Scanning.

[82] Noruzi M., Zare D., Khoshnevisan K., Davoodi D. (2011). Rapid green synthesis of gold nanoparticles using Rosa hybrida petal extract at room temperature. Spectrochim Acta Part A Mol Biomol Spectrosc..

[83] Rahimi-Nasrabadi M., Pourmortazavi S.M., Shandiz S.A.S., Ahmadi F., Batooli H. (2014). Green synthesis of silver nanoparticles using Eucalyptus leucoxylon leaves extract and evaluating the antioxidant activities of extract. Nat Prod Res.

[84] Vijayaraghavan K., Ashokkumar T. (2017). Plant-mediated biosynthesis of metallic nanoparticles: a review of literature, factors affecting synthesis, characterization techniques and applications. J Environ Chem Eng.

[85] Joshi M., Bhattacharyya A., Ali S.W. (2008). Characterization techniques for nanotechnology applications in textiles. Indian J Fibre Text Res.

[86] Eppler A.S., Rupprechter G., Anderson E.A., Somorjai G.A. (2000). Thermal and chemical stability and adhesion strength of Pt nanoparticle arrays supported on silica studied by transmission electron microscopy and atomic force microscopy. J Phys Chem B.

[87] Lange H. (1995). Comparative test of methods to determine particle size and particle size distribution in the submicron range. Part Part Syst Char.

[88] Yin I.X., Zhang J., Zhao I.S., Mei M.L., Li Q., Chu C.H. (2020). The antibacterial mechanism of silver nanoparticles and its application in dentistry. Int J Nanomed.

[89] Nie P., Zhao Y., Xu H. (2023). Synthesis, applications, toxicity and toxicity mechanisms of silver nanoparticles: a review. Ecotoxicol Environ Saf.

[90] Ratan Z.A., Haidere M.F., Nurunnabi M. (2020). Green chemistry synthesis of silver nanoparticles and their potential anticancer effects. Cancers.

[91] Palai P.K., Mondal A., Chakraborti C.K., Banerjee I., Pal K. (2019). Green synthesized amino-PEGylated silver decorated graphene nanoplatform as a tumor-targeted controlled drug delivery system. SN Appl Sci.

[92] Pistritto G., Trisciuoglio D., Ceci C., Garufi Alessia, D'Orazi G. (2016). Apoptosis as anticancer mechanism: function and dysfunction of its modulators and targeted therapeutic strategies. Aging.

[93] O'Brien M.A., Kirby R. (2008). Apoptosis: a review of pro-apoptotic and anti-apoptotic pathways and dysregulation in disease. J Vet Emerg Crit Care.

[94] Akter M., Sikder M.T., Rahman M.M. (2018). A systematic review on silver nanoparticles-induced cytotoxicity: physicochemical properties and perspectives. J Adv Res.

[95] Wu J. (2021). The enhanced permeability and retention (Epr) effect: the significance of the concept and methods to enhance its application. J Personalized Med.

[96] Liu J., Chen Q., Feng L., Liu Z. (2018). Nanomedicine for tumor microenvironment modulation and cancer treatment enhancement. Nano Today.

[97] Rónavári A., Bélteky P., Boka E. (2021). Polyvinyl-pyrrolidone-coated silver nanoparticles—the colloidal, chemical and biological consequences of steric stabilization under biorelevant conditions. Int J Mol Sci.

[98] Guo D., Zhu L., Huang Z. (2013). Anti-leukemia activity of PVP-coated silver nanoparticles via generation of reactive oxygen species and release of silver ions. Biomaterials.

[99] Palaniappan P., Sathishkumar G., Sankar R. (2015). Fabrication of nano-silver particles using Cymodocea serrulata and its cytotoxicity effect against human lung cancer A549 cells line. Spectrochim Acta Part A Mol Biomol Spectrosc..

[100] Dawadi S., Katuwal S., Gupta A. (2021). Current research on silver nanoparticles: synthesis, characterization, and applications. J Nanomater.

[101] Syed Abdul Rahman SN., Abdul Wahab N., Abd Malek S.N. (2013).

[102] Vasanth K., Ilango K., MohanKumar R., Agrawal A., Dubey G.P. (2014). Anticancer activity of Moringa oleifera mediated silver nanoparticles on human cervical carcinoma cells by apoptosis induction. Colloids Surf B Biointerfaces.

[103] Takáč P., Michalková R., Čižmáriková M., Bedlovičová Z., Balážová Ľ., Takáčová G. (2023). The role of silver nanoparticles in the diagnosis and treatment of cancer: are there any perspectives for the future?. Life.

[104] Neamati N., Fernandez A., Wright S., Kiefer J., McConkey D.J. (1995). Degradation of lamin B1 precedes oligonucleosomal DNA fragmentation in apoptotic thymocytes and isolated thymocyte nuclei. J Immunol.

[105] Redza-Dutordoir M., Averill-Bates D.A. (2016). Activation of apoptosis signalling pathways by reactive oxygen species. Biochim Biophys Acta Mol Cell Res.

[106] Levine A.J., Oren M. (2009). The first 30 years of p53: growing ever more complex. Nat Rev Cancer.

[107] Alao J.P. (2007).

[108] Johansson N., Ahonen M., Kähäri V.M. (2000). Matrix metalloproteinases in tumor invasion. Cell Mol Life Sci.

[109] Battistel D., Baldi F., Gallo M., Faleri C., Daniele S. (2015). Characterisation of biosynthesised silver nanoparticles by scanning electrochemical microscopy (SECM) and voltammetry. Talanta.

[110] Gurunathan S., Park J.H., Han J.W., Kim J.H. (2015). Comparative assessment of the apoptotic potential of silver nanoparticles synthesized by Bacillus tequilensis and Calocybe indica in MDA-MB-231 human breast cancer cells: targeting p53 for anticancer therapy. Int J Nanomed.

[111] Saratale R.G., Benelli G., Kumar G., Kim D.S., Saratale G.D. (2018). Bio-fabrication of silver nanoparticles using the leaf extract of an ancient herbal medicine, dandelion (Taraxacum officinale), evaluation of their antioxidant, anticancer potential, and antimicrobial activity against phytopathogens. Environ Sci Pollut Res.

[112] El-Naggar N.E.A., Hussein M.H., El-Sawah A.A. (2017). Bio-fabrication of silver nanoparticles by phycocyanin, characterization, in vitro anticancer activity against breast cancer cell line and in vivo cytotxicity. Sci Rep.

[113] Nagajyothi P.C., Sreekanth T.V.M., Il Lee J., Lee K.D. (2014). Mycosynthesis: antibacterial, antioxidant and antiproliferative activities of silver nanoparticles synthesized from Inonotus obliquus (Chaga mushroom) extract. J Photochem Photobiol B Biol.

[114] Krishnaraj C., Muthukumaran P., Ramachandran R., Balakumaran M.D., Kalaichelvan P.T. (2014). Acalypha indica Linn: biogenic synthesis of silver and gold nanoparticles and their cytotoxic effects against MDA-MB-231, human breast cancer cells. Biotechnol Reports.

[115] Haque S., Norbert C.C., Acharyya R., Mukherjee S., Kathirvel M., Patra C.R. (2021). Biosynthesized silver nanoparticles for cancer therapy and in vivo bioimaging. Cancers.

[116] Gurunathan S., Raman J., Abd Malek S.N., John P.A., Vikineswary S. (2013). Green synthesis of silver nanoparticles using Ganoderma neo-japonicum Imazeki: a potential cytotoxic agent against breast cancer cells. Int J Nanomed.

[117] Kuppusamy P., Ichwan S.J.A., Al-Zikri P.N.H. (2016). In vitro anticancer activity of Au, Ag nanoparticles synthesized using Commelina nudiflora L. Aqueous extract against HCT-116 colon cancer cells. Biol Trace Elem Res.

[118] Kathiravan V., Ravi S., Ashokkumar S. (2014). Synthesis of silver nanoparticles from Melia dubia leaf extract and their in vitro anticancer activity. Spectrochim Acta Part A Mol Biomol Spectrosc..

[119] Rathi Sre PR., Reka M., Poovazhagi R., Arul Kumar M., Murugesan K. (2015). Antibacterial and cytotoxic effect of biologically synthesized silver nanoparticles using aqueous root extract of Erythrina indica lam. Spectrochim Acta Part A Mol Biomol Spectrosc..

[120] Lugano R., Ramachandran M., Dimberg A. (2020). Tumor angiogenesis: causes, consequences, challenges and opportunities. Cell Mol Life Sci.

[121] Saeed B.A., Lim V., Yusof N.A., Khor K.Z., Rahman H.S., Samad N.A. (2019). Antiangiogenic properties of nanoparticles: a systematic review. Int J Nanomed.

[122] Gurunathan S., Lee K.J., Kalishwaralal K., Sheikpranbabu S., Vaidyanathan R., Eom S.H. (2009). Antiangiogenic properties of silver nanoparticles. Biomaterials.

[123] Mukherjee S., Patra C.R. (2016). Therapeutic application of anti-angiogenic nanomaterials in cancers. Nanoscale.

[124] Zhang X.F., Shen W., Gurunathan S. (2016). Silver nanoparticle-mediated cellular responses in various cell lines: an in vitro model. Int J Mol Sci.

[125] Sheikpranbabu S., Kalishwaralal K., Venkataraman D., Eom S.H., Park J., Gurunathan S. (2009). Silver nanoparticles inhibit VEGF-and IL-1β-induced vascular permeability via Src dependent pathway in porcine retinal endothelial cells. J Nanobiotechnol.

[126] Kanwar J.R., Mahidhara G., Kanwar R.K. (2011). Antiangiogenic therapy using nanotechnological-based delivery system. Drug Discov Today.

[127] Bethu M.S., Netala V.R., Domdi L., Tartte V., Janapala V.R. (2018). Potential anticancer activity of biogenic silver nanoparticles using leaf extract of Rhynchosia suaveolens: an insight into the mechanism. Artif Cells, Nanomed Biotechnol.

[128] Kalishwaralal K., Banumathi E., Pandian S.B.R.K. (2009). Silver nanoparticles inhibit VEGF induced cell proliferation and migration in bovine retinal endothelial cells. Colloids Surf B Biointerfaces.

[129] Liu Z.L., Chen H.H., Zheng L.L., Sun L.P., Shi L. (2023). Angiogenic signaling pathways and anti-angiogenic therapy for cancer. Signal Transduct Targeted Ther.

[130] Burdușel A.C., Gherasim O., Grumezescu A.M., Mogoantă L., Ficai A., Andronescu E. (2018). Biomedical applications of silver nanoparticles: an up-to-date overview. Nanomaterials.

[131] Mikhailova E.O. (2020). Silver nanoparticles: mechanism of action and probable bio-application. J Funct Biomater.

[132] Elumalai K., Srinivasan S., Shanmugam A. (2024).

[133] Alsaab H.O., Al-Hibs A.S., Alzhrani R. (2021). Nanomaterials for antiangiogenic therapies for cancer: a promising tool for personalized medicine. Int J Mol Sci.

[134] Ajaykumar A.P., Mathew A., Chandni A.P. (2023). Green synthesis of silver nanoparticles using the leaf extract of the medicinal plant, uvaria narum and its antibacterial, antiangiogenic, anticancer and catalytic properties. Antibiotics.

[135] Ribatti D. (2008). Napoleone Ferrara and the saga of vascular endothelial growth factor. Endothelium: Journal of Endothelial Cell Research.

[136] Duffy A.M., Bouchier-Hayes D.J., Harmey J.H. (2004). VEGF and Cancer.

[137] Abid M.R., Schoots I.G., Spokes K.C., Wu S.Q., Mawhinney C., Aird W.C. (2004). VEGF-mediated induction of MnSOD occurs through redox-dependent regulation of forkhead and Ikappa B/NF-kappa B. J Biol Chem.

[138] Dvorak H.F. (2002). Vascular permeability factor/vascular endothelial growth factor: a critical cytokine in tumor angiogenesis and a potential target for diagnosis and therapy. J Clin Oncol.

[139] Qian S., Wei Z., Yang W., Huang J., Yang Y., Wang J. (2022). The role of BCL-2 family proteins in regulating apoptosis and cancer therapy. Front Oncol.

[140] Carmeliet P. (2005). Angiogenesis in life, disease and medicine. Nature.

[141] Li T., Kang G., Wang T., Huang H. (2018). Tumor angiogenesis and anti-angiogenic gene therapy for cancer. Oncol Lett.

[142] Ma J., Waxman D.J. (2008).

[143] Jain P.K., Lee K.S., El-Sayed I.H., El-Sayed M.A. (2006). Calculated absorption and scattering properties of gold nanoparticles of different size, shape, and composition: applications in biological imaging and biomedicine. J Phys Chem B.

[144] Lopes-Coelho F., Martins F., Pereira S.A., Serpa J. (2021). Anti-angiogenic therapy: current challenges and future perspectives. Int J Mol Sci.

[145] Bhise N.S., Shmueli R.B., Sunshine J.C., Tzeng S.Y., Green J.J. (2011). Drug delivery strategies for therapeutic angiogenesis and antiangiogenesis. Expet Opin Drug Deliv.

[146] Albini A., Tosetti F., Li V.W., Noonan D.M., Li W.W. (2012). Cancer prevention by targeting angiogenesis. Nat Rev Clin Oncol.

[147] Yang T., Yao Q., Cao F., Liu Q., Liu B., Wang X.H. (2016). Silver nanoparticles inhibit the function of hypoxia-inducible factor-1 and target genes: insight into the cytotoxicity and antiangiogenesis. Int J Nanomed.

[148] Khandia R. (2015). Evaluation of silver nanoparticle mediated reduction of neovascularisation (angiogenesis) in chicken model. Adv Anim Vet Sci.

[149] Walia A., Yang J.F., Huang Y.H., Rosenblatt M.I., Chang J.H., Azar D.T. (2015). Endostatin's emerging roles in angiogenesis, lymphangiogenesis, disease, and clinical applications. Biochim Biophys Acta Gen Subj.

[150] Truong D.H., Le V.K.H., Pham T.T., Dao A.H., Pham T.P.D., Tran T.H. (2020). Delivery of erlotinib for enhanced cancer treatment: an update review on particulate systems. J Drug Deliv Sci Technol.

[151] Punganuru S.R., Madala H.R., Mikelis C.M., Dixit A., Arutla V., Srivenugopal K.S. (2018). Conception, synthesis, and characterization of a rofecoxibcombretastatin hybrid drug with potent cyclooxygenase-2 (COX-2) inhibiting and microtubule disrupting activities in colon cancer cell culture and xenograft models. Oncotarget.

[152] Tołoczko-Iwaniuk N., Dziemiańczyk-Pakieła D., Nowaszewska B.K., Celińska-Janowicz K., Miltyk W. (2018). Celecoxib in cancer therapy and prevention – review. Curr Drug Targets.

[153] Kong D.H., Kim M.R., Jang J.H., Na H.J., Lee S. (2017). A review of anti-angiogenic targets for monoclonal antibody cancer therapy. Int J Mol Sci.

[154] Dragovich T., Laheru D., Dayyani F. (2014). Phase II trial of vatalanib in patients with advanced or metastatic pancreatic adenocarcinoma after first-line gemcitabine therapy (PCRT O4-001). Cancer Chemother Pharmacol.

[155] Tang P.A., Moore M.J. (2013). Aflibercept in the treatment of patients with metastatic colorectal cancer: latest findings and interpretations. Therap Adv Gastroenterol.

[156] Zhang X.H., Cao M.Q., Li X.X., Zhang T. (2020). Apatinib as an alternative therapy for advanced hepatocellular carcinoma. World J Hepatol.

[157] Kotcherlakota R., Das S., Patra C.R. (2018). Green Synthesis, Characterization and Applications of Nanoparticles.

[158] Deshpande A.D., Harris-Hayes M., Schootman M. (2008). Epidemiology of diabetes and diabetes-related complications. Phys Ther.

[159] Asmat U., Abad K., Ismail K. (2016). Diabetes mellitus and oxidative stress—a concise review. Saudi Pharmaceut J.

[160] Atlas D. (2021). IDF Diabetes Atlas.

[161] Cho N.H., Shaw J.E., Karuranga S. (2018). IDF Diabetes Atlas: global estimates of diabetes prevalence for 2017 and projections for 2045. Diabetes Res Clin Pract.

[162] González P., Lozano P., Ros G., Solano F. (2023). Hyperglycemia and oxidative stress: an integral, updated and critical overview of their metabolic interconnections. Int J Mol Sci.

[163] Romaniyanto Mahyudin F., Sigit Prakoeswa C.R., Notobroto H.B. (2022). An update of current therapeutic approach for Intervertebral Disc Degeneration: a review article. Annals of Medicine and Surgery.

[164] Davies M.J., Aroda V.R., Collins B.S. (2022). Management of hyperglycemia in type 2 diabetes, 2022. A consensus report by the American diabetes association (ada) and the European association for the study of diabetes (easd). Diabetes Care.

[165] Malik S., Muhammad K., Waheed Y. (2023). Nanotechnology: a revolution in modern industry. Molecules.

[166] Lemmerman L.R., Das D., Higuita-Castro N., Mirmira R.G., Gallego-Perez D. (2020). Nanomedicine-based strategies for diabetes: diagnostics, monitoring, and treatment. Trends Endocrinol Metabol.

[167] Kaushal A., Khurana I., Yadav P. (2023). Advances in therapeutic applications of silver nanoparticles. Chem Biol Interact.

[168] Selvaraj K., Arthanari A., Rajeshkumar S. (2023). Biosynthesis of silver nanoparticles using rose and jasmine and its anti diabetic potential. J Complement Med Res.

[169] Wahab M., Bhatti A., John P. (2022). Evaluation of antidiabetic activity of biogenic silver nanoparticles using thymus serpyllum on streptozotocin-induced diabetic BALB/c mice. Polymers.

[170] Sengani M., V B., Banerjee M. (2022). Evaluation of the anti-diabetic effect of biogenic silver nanoparticles and intervention in PPARγ gene regulation. Environ Res.

[171] Bedlovičová Z., Strapáč I., Baláž M., Salayová A. (2020). A brief overview on antioxidant activity determination of silver nanoparticles. Molecules.

[172] Giacco F., Brownlee M. (2010). Oxidative stress and diabetic complications. Circ Res.

[173] Ashwini D., Mahalingam G. (2020).

[174] Gong L., Feng D., Wang T., Ren Y., Liu Y., Wang J. (2020). Inhibitors of α-amylase and α-glucosidase: potential linkage for whole cereal foods on prevention of hyperglycemia. Food Sci Nutr.

[175] Saratale R.G., Shin H.S., Kumar G., Benelli G., Kim D.S., Saratale G.D. (2018). Exploiting antidiabetic activity of silver nanoparticles synthesized using punica granatum leaves and anticancer potential against human liver cancer cells (HepG2). Artif Cells, Nanomed Biotechnol.

[176] Sati S.C., Kour G., Bartwal A.S., Sati M.D. (2020). Biosynthesis of metal nanoparticles from leaves of Ficus palmata and evaluation of their anti-inflammatory and anti-diabetic activities. Biochemistry.

[177] Balan K., Qing W., Wang Y. (2016). Antidiabetic activity of silver nanoparticles from green synthesis using Lonicera japonica leaf extract. RSC Adv.

[178] Saratale R.G., Saratale G.D., Ahn S., Shin H.S. (2021). Grape pomace extracted tannin for green synthesis of silver nanoparticles: assessment of their antidiabetic, antioxidant potential and antimicrobial activity. Polymers.

[179] Garcia-Campoy A., Garcia E., Muñiz-Ramirez A. (2020). Phytochemical and pharmacological study of the eysenhardtia genus. Plants.

[180] Olugbodi J.O., Lawal B., Bako G. (2023). Effect of sub-dermal exposure of silver nanoparticles on hepatic, renal and cardiac functions accompanying oxidative damage in male Wistar rats. Sci Rep.

[181] Graham M.L., Janecek J.L., Kittredge J.A., Hering B.J., Schuurman H.J. (2011). The streptozotocin-induced diabetic nude mouse model: differences between animals from different sources. Comp Med.

[182] Zang L., Shimada Y., Nishimura N. (2017). Development of a novel zebrafish model for type 2 diabetes mellitus. Sci Rep.

[183] Fu Z., Gilbert E R., Liu D. (2012). Regulation of insulin synthesis and secretion and pancreatic beta-cell dysfunction in diabetes. Curr Diabetes Rev.

[184] Gutierrez R.M.P., Jeronimo F.F.M., Campoy A.H.G., Vadillo C.H. (2018). Silver nanoparticles synthesized using eysenhardtia polystachya and assessment of the inhibition of glycation in multiple stages in vitro and in the zebrafish model. J Cluster Sci.

[185] Choudhury H., Pandey M., Lim Y.Q. (2020). Silver nanoparticles: advanced and promising technology in diabetic wound therapy. Mater Sci Eng C.

[186] Matough F.A., Budin S.B., Hamid Z.A., Alwahaibi N., Mohamed J. (2012). The role of oxidative stress and antioxidants in diabetic complications. Sultan Qaboos University Medical Journal.

[187] Wong K.K.Y., Cheung S.O.F., Huang L. (2009). Further evidence of the anti-inflammatory effects of silver nanoparticles. ChemMedChem.

[188] Jini D., Sharmila S., Anitha A., Pandian M., Rajapaksha R.M.H. (2022). In vitro and in silico studies of silver nanoparticles (AgNPs) from Allium sativum against diabetes. Sci Rep.

[189] Boucher J., Kleinridders A., Ronald Kahn C. (2014). Insulin receptor signaling in normal and insulin-resistant states. Cold Spring Harbor Perspect Biol.

[190] Percival S.L., Suleman L., Vuotto C., Donelli G. (2015). Healthcare-Associated infections, medical devices and biofilms: risk, tolerance and control. J Med Microbiol.

[191] Lindholm C., Searle R. (2016). Wound management for the 21st century: combining effectiveness and efficiency. Int Wound J.

[192] Hsu J.T., Chen Y.W., Ho T.W. (2019). Chronic wound assessment and infection detection method. BMC Med Inf Decis Making.

[193] Järbrink K., Ni G., Sönnergren H. (2017). The humanistic and economic burden of chronic wounds: a protocol for a systematic review. Syst Rev.

[194] Singh S., Young A., McNaught C.E. (2017). The physiology of wound healing. Surgery.

[195] Schultz G.S., Chin G.A., Moldawer L., Diegelmann R.F. (2011). Mechanisms of Vascular Disease: A Reference Book for Vascular Specialists.

[196] Gonzalez A.C.D.O., Andrade Z.D.A., Costa T.F., Medrado A.R.A.P. (2016). Wound healing - a literature review. An Bras Dermatol.

[197] Han G., Ceilley R. (2017). Chronic wound healing: a review of current management and treatments. Adv Ther.

[198] Gould L., Abadir P., Brem H. (2015). Chronic wound repair and healing in older adults: current status and future research. Wound Repair Regen.

[199] Sen C.K., Gordillo G.M., Roy S. (2009). Human skin wounds: a major and snowballing threat to public health and the economy: perspective article. Wound Repair Regen.

[200] Powers J.G., Higham C., Broussard K., Phillips T.J. (2016). Wound healing and treating wounds Chronic wound care and management. J Am Acad Dermatol.

[201] Nayak B.S., Sandiford S., Maxwell A. (2009). Evaluation of the wound-healing activity of ethanolic extract of Morinda citrifolia L. leaf. Evid base Compl Alternative Med.

[202] Dissemond J., Böttrich J.G., Braunwarth H., Hilt J., Wilken P., Münter K.C. (2017). Evidence for silver in wound care – meta-analysis of clinical studies from 2000–2015. JDDG - J Ger Soc Dermatology.

[203] Kumar S.S.D., Rajendran N.K., Houreld N.N., Abrahamse H. (2018). Recent advances on silver nanoparticle and biopolymer-based biomaterials for wound healing applications. Int J Biol Macromol.

[204] Möhler J.S., Sim W., Blaskovich M.A.T., Cooper M.A., Ziora Z.M. (2018). Silver bullets: a new lustre on an old antimicrobial agent. Biotechnol Adv.

[205] Dai X., Guo Q., Zhao Y. (2016). Functional silver nanoparticle as a benign antimicrobial agent that eradicates antibiotic-resistant bacteria and promotes wound healing. ACS Appl Mater Interfaces.

[206] Cameron S.J., Hosseinian F., Willmore W.G. (2018). A current overview of the biological and cellular effects of nanosilver. Int J Mol Sci.

[207] Chauhan P.S., Shrivastava V., Gbks P., Tomar R.S. (2018). Effect of silver nanoparticle-mediated wound therapy on biochemical, hematological, and histological parameters. Asian J Pharmaceut Clin Res.

[208] Rigo C., Ferroni L., Tocco I. (2013). Active silver nanoparticles for wound healing. Int J Mol Sci.

[209] Ahmadi M., Adibhesami M. (2017). The effect of silver nanoparticles on wounds contaminated with Pseudomonas aeruginosa in mice: an experimental study. Iran J Pharm Res (IJPR).

[210] Rujitanaroj P on, Pimpha N., Supaphol P. (2008). Wound-dressing materials with antibacterial activity from electrospun gelatin fiber mats containing silver nanoparticles. Polymer.

[211] Miller C.N., Newall N., Kapp S.E. (2010). A randomized-controlled trial comparing cadexomer iodine and nanocrystalline silver on the healing of leg ulcers. Wound Repair Regen.

[212] Parveen A., Kulkarni N., Yalagatti M., Abbaraju V., Deshpande R. (2018). In vivo efficacy of biocompatible silver nanoparticles cream for empirical wound healing. J Tissue Viability.

[213] Diller R.B., Tabor A.J. (2022). The role of the extracellular matrix (ECM) in wound healing: a review. Biomimetics.

[214] Nakamura T., Kambayashi J.I., Okuma M., Tandon N.N. (1999). Activation of the GP IIb-IIIa complex induced by platelet adhesion to collagen is mediated by both α2β1 integrin and GP VI. J Biol Chem.

[215] Pierce G.F., Mustoe T.A., Lingelbach J. (1989). Platelet-derived growth factor and transforming growth factor-β enhance tissue repair activities by unique mechanisms. J Cell Biol.

[216] Martinez-Zapata M.J., Martí-Carvajal A.J., Solà I. (2016). Autologous platelet-rich plasma for treating chronic wounds. Cochrane Database Syst Rev.

[217] Paladini F., Pollini M. (2019). Antimicrobial silver nanoparticles for wound healing application: progress and future trends. Materials.

[218] Elkington P.T.G., Friedland J.S. (2006). Matrix metalloproteinases in destructive pulmonary pathology. Thorax.

[219] Nqakala Z.B., Sibuyi N.R.S., Fadaka A.O., Meyer M., Onani M.O., Madiehe A.M. (2021). Advances in nanotechnology towards development of silver nanoparticle-based wound-healing agents. Int J Mol Sci.

[220] Khalil M.M.H., Ismail E.H., El-Baghdady K.Z., Mohamed D. (2014). Green synthesis of silver nanoparticles using olive leaf extract and its antibacterial activity. Arab J Chem.

[221] Mubeen B., Ansar A.N., Rasool R. (2021). Nanotechnology as a novel approach in combating microbes providing an alternative to antibiotics. Antibiotics.

[222] Dakal T.C., Kumar A., Majumdar R.S., Yadav V. (2016). Mechanistic basis of antimicrobial actions of silver nanoparticles. Front Microbiol.

[223] More P.R., Pandit S., Filippis A De, Franci G., Mijakovic I., Galdiero M. (2023). Silver nanoparticles: bactericidal and mechanistic approach against drug resistant pathogens. Microorganisms.

[224] Du J., Hu Z., Yu Z. (2019). Antibacterial activity of a novel Forsythia suspensa fruit mediated green silver nanoparticles against food-borne pathogens and mechanisms investigation. Mater Sci Eng C.

[225] Mukunthan K.S., Elumalai E.K., Patel T.N., Murty V.R. (2011). Catharanthus roseus: a natural source for the synthesis of silver nanoparticles. Asian Pac J Trop Biomed.

[226] Gugala N., Vu D., Parkins M.D., Turner R.J. (2019). Specificity in the susceptibilities of Escherichia coli, Pseudomonas aeruginosa and Staphylococcus aureus clinical isolates to six metal antimicrobials. Antibiotics.

[227] Shahverdi A.R., Minaeian S., Shahverdi H.R., Jamalifar H., Nohi A.A. (2007). Rapid synthesis of silver nanoparticles using culture supernatants of Enterobacteria: a novel biological approach. Process Biochem.

[228] Nanda A., Saravanan M. (2009). Biosynthesis of silver nanoparticles from Staphylococcus aureus and its antimicrobial activity against MRSA and MRSE. Nanomed Nanotechnol Biol Med.

[229] Zhou J., Cai Y., Liu Y. (2022). Breaking down the cell wall: still an attractive antibacterial strategy. Front Microbiol.

[230] Quinteros M.A., Viviana C.A., Onnainty R. (2018). Biosynthesized silver nanoparticles: decoding their mechanism of action in Staphylococcus aureus and Escherichia coli. Int J Biochem Cell Biol.

[231] Qing Y., Cheng L., Li R. (2018). Potential antibacterial mechanism of silver nanoparticles and the optimization of orthopedic implants by advanced modification technologies. Int J Nanomed.

[232] Anees Ahmad S., Sachi Das S., Khatoon A. (2020). Bactericidal activity of silver nanoparticles: a mechanistic review. Materials Science for Energy Technologies.

[233] Durán N., Nakazato G., Seabra A.B. (2016). Antimicrobial activity of biogenic silver nanoparticles, and silver chloride nanoparticles: an overview and comments. Appl Microbiol Biotechnol.

[234] Selem E., Mekky A.F., Hassanein W.A., Reda F.M., Selim Y.A. (2022). Antibacterial and antibiofilm effects of silver nanoparticles against the uropathogen Escherichia coli U12. Saudi J Biol Sci.

[235] Arif R., Uddin R. (2021). A review on recent developments in the biosynthesis of silver nanoparticles and its biomedical applications. Med DEVICES SENSORS..

[236] Wang L., Hu C., Shao L. (2017). The antimicrobial activity of nanoparticles: present situation and prospects for the future. Int J Nanomed.

[237] Sharmin S., Rahaman M.M., Sarkar C., Atolani O., Islam M.T., Adeyemi O.S. (2021). Nanoparticles as antimicrobial and antiviral agents: a literature-based perspective study. Heliyon.

[238] Li W.R., Xie X.B., Shi Q.S., Zeng H.Y., Ou-Yang Y.S., Chen Y Ben (2010). Antibacterial activity and mechanism of silver nanoparticles on Escherichia coli. Appl Microbiol Biotechnol.

[239] Elsawy S., Elsherif W.M., Hamed R. (2021). Effect of silver nanoparticles on vancomycin resistant Staphylococcus aureus infection in critically ill patients. Pathog Glob Health.

[240] Loo Y.Y., Rukayadi Y., Nor-Khaizura M.A.R. (2018). In Vitro antimicrobial activity of green synthesized silver nanoparticles against selected Gram-negative foodborne pathogens. Front Microbiol.

[241] Tran H.M., Tran H., Booth M.A. (2020). Nanomaterials for treating bacterial biofilms on implantable medical devices. Nanomaterials.

[242] Naumenko K., Zahorodnia S., Pop C.V., Rizun N. (2023). Antiviral activity of silver nanoparticles against the influenza A virus. J Virus Erad.

[243] Rai M., Yadav A., Gade A. (2009). Silver nanoparticles as a new generation of antimicrobials. Biotechnol Adv.

[244] Mori Y., Ono T., Miyahira Y., Nguyen V.Q., Matsui T., Ishihara M. (2013). Antiviral activity of silver nanoparticle/chitosan composites against H1N1 influenza A virus. Nanoscale Res Lett.

[245] Etemadzade M., Ghamarypour A., Zabihollahi R. (2016). Synthesis and evaluation of antiviral activities of novel sonochemical silver nanorods against HIV and HSV viruses. Asian Pacific J Trop Dis.

[246] Gaikwad S., Ingle A., Gade A. (2013). Antiviral activity of mycosynthesized silver nanoparticles against herpes simplex virus and human parainfluenza virus type 3. Int J Nanomed.

[247] Lu L., Sun R.W.Y., Chen R. (2008). Silver nanoparticles inhibit hepatitis B virus replication. Antivir Ther.

[248] Aderibigbe B.A. (2017). Metal-based nanoparticles for the treatment of infectious diseases. Molecules.

[249] Luceri A., Francese R., Lembo D., Ferraris M., Balagna C. (2023). Silver nanoparticles: review of antiviral properties, mechanism of action and applications. Microorganisms.

[250] He Q., Lu J., Liu N. (2022). Antiviral properties of silver nanoparticles against SARS-CoV-2: effects of surface coating and particle size. Nanomaterials.

[251] Galdiero S., Falanga A., Vitiello M., Cantisani M., Marra V., Galdiero M. (2011). Silver nanoparticles as potential antiviral agents. Molecules.

[252] Sarkar D.S. (2020). Silver nanoparticles with bronchodilators through nebulisation to treat COVID 19 patients. J Curr Med Res Opin.

[253] Lara H.H., Ayala-Nuñez N.V., Ixtepan-Turrent L., Rodriguez-Padilla C. (2010). Mode of antiviral action of silver nanoparticles against HIV-1. J Nanobiotechnol.

[254] Gurunathan S., Qasim M., Choi Y. (2020). Antiviral potential of nanoparticles—can nanoparticles fight against coronaviruses?. Nanomaterials.

[255] Lin N., Verma D., Saini N. (2021). Antiviral nanoparticles for sanitizing surfaces: a roadmap to self-sterilizing against COVID-19. Nano Today.

[256] Morris D., Ansar M., Speshock J. (2019). Antiviral and immunomodulatory activity of silver nanoparticles in experimental rsv infection. Viruses.

[257] Longhi C., Santos J.P., Morey A.T. (2016). Combination of fluconazole with silver nanoparticles produced by Fusarium oxysporum improves antifungal effect against planktonic cells and biofilm of drug-resistant Candida albicans. Med Mycol.

[258] Speshock J.L., Murdock R.C., Braydich-Stolle L.K., Schrand A.M., Hussain S.M. (2010). Interaction of silver nanoparticles with Tacaribe virus. J Nanobiotechnol.

[259] Jalal R., Goharshadi E.K., Abareshi M., Moosavi M., Yousefi A., Nancarrow P. (2010). ZnO nanofluids: green synthesis, characterization, and antibacterial activity. Mater Chem Phys.

[260] Ogar A., Tylko G., Turnau K. (2015). Antifungal properties of silver nanoparticles against indoor mould growth. Sci Total Environ.

[261] Carmo PHF do, Garcia M.T., Figueiredo-Godoi L.M.A., Lage A.C.P., Silva NS da, Junqueira J.C. (2023). Metal nanoparticles to combat Candida albicans infections: an update. Microorganisms.

[262] Singh J., Vishwakarma K., Ramawat N. (2019). Nanomaterials and microbes' interactions: a contemporary overview. 3 Biotech..

[263] Mallmann E.J.J., Cunha F.A., Castro B.N.M.F., Maciel A.M., Menezes E.A., Fechine P.B.A. (2015). Antifungal activity of silver nanoparticles obtained by green synthesis. Rev Inst Med Trop Sao Paulo.

[264] Jalal M., Ansari M.A., Alzohairy M.A. (2018). Biosynthesis of silver nanoparticles from oropharyngeal candida glabrata isolates and their antimicrobial activity against clinical strains of bacteria and fungi. Nanomaterials.

[265] Jalal M., Ansari M.A., Alzohairy M.A. (2019). Anticandidal activity of biosynthesized silver nanoparticles: effect on growth, cell morphology, and key virulence attributes of Candida species. Int J Nanomed.

[266] Mohsen L.Y., Fadhil Alsaffar M., Ahmed Lilo R., Khalil Al-Shamari A. (2022). Silver nanoparticles that synthesis by using Trichophyton rubrum and evaluate antifungal activity. Arch Razi Inst.

[267] Ndikau M., Noah N.M., Andala D.M., Masika E. (2017). Green synthesis and characterization of silver nanoparticles using Citrullus lanatus fruit rind extract. Int J Anal Chem.

[268] Salem S.S., Ali O.M., Reyad A.M., Abd-Elsalam K.A., Hashem A.H. (2022). Pseudomonas indica-mediated silver nanoparticles: antifungal and antioxidant biogenic tool for suppressing mucormycosis fungi. J Fungi.

[269] Datye A., DeLaRiva A. (2023).

[270] Ali E.M., Abdallah B.M. (2022). Effective inhibition of invasive pulmonary aspergillosis by silver nanoparticles biosynthesized with artemisia sieberi leaf extract. Nanomaterials.

[271] Wen H., Shi H., Jiang N., Qiu J., Lin F., Kou Y. (2023). Antifungal mechanisms of silver nanoparticles on mycotoxin producing rice false smut fungus. iScience.

[272] Panáček A., Kolář M., Večeřová R. (2009). Antifungal activity of silver nanoparticles against Candida spp. Biomaterials.

